# Pediatricians’ attitudes and knowledge of RSV immunization products: A multi-country cross-sectional survey

**DOI:** 10.1371/journal.pone.0356233

**Published:** 2026-08-17

**Authors:** Costanza Di Chiara, Pierre-Philippe Piché-Renaud, Vera Rigamonti, Anna Cantarutti, Cristina Calvo, Irene Rivero Calle, Antoni Soriano-Arandes, Carlo Giaquinto, Luigi Cantarutti, Daniele Donà, Andrea Lo Vecchio, Adamos Hadjipanayis, Zoi Dorothea Pana, Ioannis Kopsidas, Adria Rose, Alfredo Tagarro, Shaun K. Morris

**Affiliations:** 1 Child Health Evaluative Sciences, The Hospital for Sick Children, Toronto, Ontario, Canada; 2 Division of Infectious Diseases, The Hospital for Sick Children, Toronto, Ontario, Canada; 3 Department of Women’s and Children’s Health, University of Padova, Padova, Italy; 4 Department of Pediatrics, Faculty of Medicine, University of Toronto, Toronto, Ontario, Canada; 5 Laboratory of Healthcare Research and Pharmacoepidemiology, Division of Biostatistics, Epidemiology and Public Health, Department of Statistic and Quantitative Methods, University of Milano-Bicocca, Milano, Italy; 6 Pediatric Infectious Diseases Department, La Paz University Hospital, Madrid, Spain; 7 Hospital La Paz Institute for Health Research (IdiPAZ), Madrid, Spain; 8 Translational Research Network in Pediatric Infectious Diseases (RITIP), Madrid, Spain; 9 CIBER de Enfermedades Infecciosas, CIBERINFEC, ISCIII, Madrid, Spain; 10 Translational Pediatrics and Infectious Diseases, Hospital Clínico Universitario de Santiago (SERGAS) and University of Santiago de Compostela (USC), Santiago de Compostela, Galicia, Spain; 11 Genetics, Vaccines and Infectious Diseases Research Group (GENVIP), Instituto de Investigación Sanitaria de Santiago de Compostela (IDIS), Santiago de Compostela, Galicia, Spain; 12 WHO Collaborating Centre for Vaccine Safety, Santiago de Compostela, Galicia, Spain; 13 Centro de Investigación Biomédica en Red de Enfermedades Respiratorias (CIBERES), Instituto de Salud Carlos III, Madrid, Spain; 14 Department of Paediatrics, Sistemes de Salut Integrats del Baix Empordà (SSIBE), Girona, Catalonia, Spain; 15 Infection and Immunity in Paediatric Patients, Vall d’Hebron Research Institute, Barcelona, Catalonia, Spain; 16 Società Servizi Telematici-Pedianet, Padova, Italy; 17 Department of Translational Medical Science - Section of Paediatrics, University of Naples Federico II, Naples, Italy; 18 Medical School, European University of Cyprus, Nicosia, Cyprus; 19 Department of Clinical and Basic Studies, Medical School, European University of Cyprus, Nicosia, Cyprus; 20 Center for Clinical Epidemiology and Outcomes Research, Department of Pediatrics, School of Medicine, National and Kapodistrian University of Athens, Athens, Greece; 21 Paediatrics Department, Hospital Universitario Infanta Sofía, Paediatrics Research Group, Universidad Europea de Madrid, Madrid, Spain; 22 Fundación de Investigación Biomédica Hospital 12 de Octubre, Instituto de Investigación 12 de Octubre (imas12), Madrid, Spain; 23 Fundación para la Investigación Biomédica Hospital Infanta Sofía y del Henares, Madrid, Spain; 24 Division of Clinical Public Health and Centre for Vaccine Preventable Diseases, Dalla Lana School of Public Health, Toronto, Ontario, Canada; Federal University of Sergipe, BRAZIL

## Abstract

**Objective:**

Nirsevimab and the maternal vaccination in pregnancy are newly introduced prevention strategies against respiratory syncytial virus (RSV). This study evaluated pediatricians’ knowledge, attitudes, and barriers to RSV immunization in Italy and Cyprus/Greece, where nirsevimab had not yet been implemented, and in Spain, where it was introduced in 2023/2024.

**Methods:**

A survey was distributed to pediatricians across Spain, Italy, and Cyprus/Greece in July-August/2024. Occupational characteristics, and knowledge, attitudes and practices toward palivizumab, nirsevimab, and the maternal vaccine were collected. Of 578 respondents (response rate = 19.3%), 50% were from Spain, 40% from Italy, and 10% from Cyprus/Greece. As 90% of responses came from Spain and Italy, the main analysis focused on these two countries. Descriptive and univariate analyses (Chi-square or Fisher’s exact tests) were conducted.

**Results:**

Respondents included pediatricians from community (51.7%), tertiary (30.4%), and primary/secondary care (17.9%) settings. Pediatricians were generally aware of RSV risk factors, without differences across countries. However, 245 (55.9%) and 375 (86.0%) participants reported average-to-low knowledge of nirsevimab and the maternal vaccine, respectively, with differences across countries (p = 0.002). Despite limited knowledge, 311 (85.9%) pediatricians were willing to administer nirsevimab, and 319 (92.2%) supported its use for all infants. Nirsevimab administration’s barriers, including unfamiliarity and logistical challenges, were cited by 69 (20.0%) pediatricians. Regarding immunization preferences, 165 (47.4%) pediatricians were very likely to support the maternal vaccine over nirsevimab.

**Conclusions:**

While pediatricians support RSV immunization, knowledge gaps and logistical barriers may hinder early-stage implementation. Optimizing education on RSV immunization will be crucial to improve uptake and prevention strategies.

## Introduction

Respiratory syncytial virus (RSV) is a leading cause of pediatric lower respiratory infections, resulting in significant hospitalizations even among healthy, term infants [1–5]. Historically, palivizumab, a monthly monoclonal antibody, was the only available RSV prophylaxis, but its high cost limited use to high-risk infants [6,7].

In 2023, the European Medicines Agency (EMA) and the U.S. Food and Drug Administration (FDA) approved nirsevimab, a long-acting monoclonal antibody targeting the highly conserved epitope site Ø on the prefusion form of the RSV fusion (F) protein. Administered as a single dose to all infants during their first RSV season, it demonstrated over 70% efficacy in preventing RSV-related lower respiratory tract infections in both preterm and term infants [8,9], with real-world data showing >80% efficacy in reducing hospitalizations and 85% against severe infections [10–18]. Additionally, both the EMA and FDA approved a maternal RSV vaccine targeting the preF protein, administered in late pregnancy to provide passive immunity to infants. However, it showed a lower efficacy (57%) against severe RSV infection in clinical trials [19].

RSV burden and new preventative tools emphasize the importance of widespread immunization, which relies on healthcare providers, particularly pediatricians, to guide caregivers’ decisions [20–24]. Therefore, understanding pediatricians’ knowledge and attitudes toward RSV and its new immunization strategies is essential for effective implementation [25]. This study assessed pediatricians’ awareness, attitudes, knowledge, and practices regarding RSV immunization in Italy, Cyprus, and Greece – countries where nirsevimab had not yet been adopted –, and in Spain, a country with active national recommendations and nirsevimab implementation during the 2023–2024 season. It also compared these factors between countries with and without access to the product.

## Methods

### Study design, setting, and data collection

A multi-language, self-administered, cross-sectional survey was conducted among pediatricians in Spain, Italy, Cyprus, and Greece between July 20 and August 24, 2024. We hypothesized that higher product knowledge and confidence in RSV immunization would correlate with stronger acceptance and greater intent to implement nirsevimab and maternal RSV vaccination in clinical practice, highlighting the role of education and implementation readiness in early adoption of new immunization strategies. The survey was electronically distributed to 2,000 members of the Spanish Pediatric Association (AEP) (including 346 members of the Spanish Society of Pediatric Infectious Diseases [SEIP]), 180 members of the Italian Society of Pediatric Infectious Diseases, 120 members of the Italian independent network of family pediatricians (Pedianet, Società Servizi Telematici Srl), 250 members of the Cypriot Pediatric Society, and 100 members of the Hellenic Society of Pediatric Infectious Diseases. Each society sent the survey via secure email listservs, with an initial invitation followed by four weekly reminders. The questionnaire was available in Spanish, Italian, Greek, and English.

### Survey instrument

The survey instrument consisted of binary, multiple-choice, and Likert-scale questions. It was originally developed by the Initiative for Vaccine Acceptance and Confidence (IVAC) at the Hospital for Sick Children in Toronto, Canada. It was then pilot-tested by general and infectious disease pediatricians to improve clarity, and subsequently adapted and translated by European researchers within the study team to ensure contextual relevance for each country. To reflect local implementation timelines, questions about palivizumab were asked in the present tense for Italy, Cyprus, and Greece (where it was still in use), and in the past tense for Spain (where nirsevimab had replaced it). Questions about nirsevimab followed the opposite approach.

At the time of the survey distribution, national recommendations on infant RSV immunization were available only in Spain. The Advisory Committee on Vaccines (CAV) of the Spanish Pediatric Association (AEP) had published its 2024 immunization guidelines, recommending the routine administration of a single dose of nirsevimab for all infants under 6 months of age and an annual dose for high-risk children under 2 years [26]. Additionally, the 2024 CAV-AEP immunization calendar advised administering a dose of maternal RSV vaccination between weeks 24 and 36 of gestation, preferably between weeks 32 and 36, as part of a public health strategy if indicated [26]. Spain’s Ministry of Health prioritized nirsevimab over maternal RSV vaccination [27]. In contrast, in Italy, only a position paper on maternal RSV vaccination during pregnancy had been published by Italian societies of gynecologists and obstetricians [28], while in Greece and Cyprus, no national recommendations on either nirsevimab or maternal RSV vaccination were available at the time of the survey.

The questionnaire included four main sections: 1) pediatricians’ occupational and demographic information, 2) knowledge and perceptions about RSV infection and prevention products, 3) attitude and practices toward palivizumab and nirsevimab, and 4) perspectives on RSV maternal vaccination. The full survey is detailed in Supplemental Materials (S1 File).

### Statistical analysis

Descriptive statistics summarized participants’ RSV knowledge, attitudes, and practices. Frequencies and percentages were calculated for each question. It was not required for all questions to be answered by the respondents in order to submit the survey. Incomplete surveys were retained in the analysis, with missing responses being excluded from question-specific calculations of frequencies and percentages.

Specific scores were developed to assess variations in RSV knowledge among pediatricians’ subgroups. These scores were constructed using a core set of key variables and designed to assess two dimensions: (1) knowledge of RSV risk factors and (2) knowledge of RSV prevention strategies. Higher scores indicated greater knowledge. The risk factor knowledge score (score #1) combined two components: identification of individual conditions associated with severe RSV disease (maximum 9 points, with penalties for incorrect responses) and identification of the population group accounting for the largest proportion of severe cases (1 point if correct), yielding a total score ranging from 5 to 10. The prevention knowledge score (score #2) was based on self-reported knowledge of three interventions (palivizumab, nirsevimab, and maternal vaccination), each rated on a 1–5 Likert scale, with a total score ranging from 3 to 15. Both scores were standardized to a 0–10 scale and categorized into three levels (low, medium, high) based on the 25th and 75th percentiles of their distribution to reflect relative differences within the study population. Additional details on score construction are provided in Supplemental Materials (S2 File). The construction of the scores was guided by content validity and clinical relevance, and formal internal consistency measures were not considered appropriate given the structure and purpose of the scores. Comparisons of knowledge levels between participant subgroups (country, subspecialty, years of practice, primary practice setting, experience managing RSV cases, and their center’s involvement in vaccination administration) was performed using Chi-square test. Scores were computed exclusively for participants who answered all the key questions necessary for their calculation (hereafter referred to as “having completed the survey”). Analyses were therefore restricted to this group, while minor missing data in secondary or optional variables were retained to avoid unnecessary reduction of the sample size. To further investigate associations between pediatrician characteristics and the main outcomes, multivariate analyses were conducted. Specifically, ordinal logistic regression models were fitted for awareness (Score 1) and attitudes (Score 2), both treated as ordered categorical variables (low, medium, high). Covariates included in the models were country, pediatrician profile, years in practice, primary practice setting, provision of immunizations, and frequency of bronchiolitis management. Covariates were selected a priori based on clinical relevance and prior literature on factors associated with knowledge and attitudes toward immunization. Results are reported as adjusted odds ratios (aORs) with 95% confidence intervals (CIs).

All statistical analyses were performed using Microsoft Excel software version 2409 and SAS software version 9.4 (SAS Institute, Inc., Cary, NC, USA). Significance was set at P < 0.05.

### Ethics

As part of a broader research initiative conducted in Canada, this study was submitted to and approved by the Hospital for Sick Children’s Research Ethics Board (REB # 1000081077), with protocol acknowledgment in Cyprus and Greece. In Spain and Italy, formal ethical approval was waived in accordance with national regulations due to the fully anonymous nature of the survey. Participation was voluntary, with implied consent upon survey completion. Surveys were completed using the Research Electronic Data Capture (REDCap) software. No identifiable personal data were collected, and the survey platform was configured to ensure participant anonymity by not recording IP addresses or other identifying information.

## Results

### Participants’ characteristics

After excluding surveys that were only opened (N = 56) or only contained country-related responses (country of training and country of practice) (N = 69), 578 participants were included (response rate = 19.3%). Spain contributed 290 (50%) participants (response rate: 14.5%), Italy 228 (40%) (response rate: 76%), Cyprus 43 (7%) (response rate: 17.2%), and Greece 17 (3%) (response rate: 17%) (Table 1). During the study period, only Spain had implemented nirsevimab at the national level, while the other countries were in a pre-implementation phase.

**Table 1:** Characteristics of respondents (N = 578).

Characteristics, N (%)	Overall (N = 578)	Spain (N = 290)	Italy (N = 228)	Cyprus (N = 43)	Greece (N = 17)
Pediatric training	***(missing = 4)***	***(missing = 1)***	***(missing = 3)***	***(missing = 0)***	***(missing = 0)***
General pediatrics	356 (62.0%)	181 (62.6%)	128 (56.9%)	40 (93.0%)	7 (41.2%)
Subspecialty pediatrics	218 (38.0%)	108 (37.4%)	97 (43.1%)	3 (7.0%)	10 (58.8%)
Primary practice setting	***(missing = 5)***	***(missing = 1)***	***(missing = 1)***	***(missing = 2)***	***(missing = 1)***
Urban	459 (80.1%)	238 (82.4%)	172 (75.8%)	34 (82.9%)	15 (93.8%)
Suburban	64 (11.2%)	26 (9.0%)	35 (15.4%)	3 (7.3%)	0 (0.0%)
Rural/remote	50 (8.7%)	25 (8.7%)	20 (8.8%)	4 (9.8%)	1 (6.3%)
Type of practice site	***(missing = 3)***	***(missing = 2)***	***(missing = 0)***	***(missing = 1)***	***(missing = 0)***
Community-based pediatricians	297 (51.7%)	145 (50.3%)	130 (57.0%)	18 (42.9%)	4 (23.5%)
Primary/secondary care hospital (public and private)	103 (17.9%)	41 (14.2%)	41 (18.0%)	19 (45.2%)	2 (11.8%)
Academic/Tertiary care hospital	175 (30.4%)	102 (35.4%)	57 (25.0%)	5 (11.9%)	11 (64.7%)
Years of practice	***(missing = 6)***	***(missing = 3)***	***(missing = 3)***	***(missing = 0)***	***(missing = 0)***
< / = 5 years	86 (15.0%)	38 (13.2%)	46 (20.4%)	1 (2.3%)	1 (5.9%)
6–10 years	64 (11.2%)	37 (12.9%)	24 (10.7%)	0 (0.0%)	3 (17.6%)
11–25 years	180 (31.5%)	112 (39.0%)	48 (21.3%)	12 (27.9%)	8 (47.1%)
> 25 years	242 (42.3%)	100 (34.8%)	107 (47.6%)	30 (7.0%)	5 (29.4%)
Pediatricians’ practices in managing cases of bronchiolitis	***(missing = 2)***	***(missing = 0)***	***(missing = 0)***	***(missing = 2)***	***(missing = 0)***
Always	277 (48.1%)	151 (52.1%)	104 (45.6%)	18 (43.9%)	4 (23.5%)
Very often	253 (43.9%)	118 (40.7%)	107 (46.9%)	18 (43.9%)	10 (58.8%)
Sometimes	40 (6.9%)	19 (6.6%)	14 (6.1%)	5 (12.2%)	2 (11.7%)
Rarely	5 (0.9%)	2 (0.7%)	2 (0.9%)	0 (0.0%)	1 (5.9%)
Never	1 (0.2%)	0 (0.0%)	1 (0.4%)	0 (0.0%)	0 (0.0%)
Working in a practice which provides immunizations	***(missing = 4)***	***(missing = 2)***	***(missing = 0)***	***(missing = 2)***	***(missing = 0)***
Yes	496 (86.4%)	260 (90.3%)	183 (80.3%)	39 (95.1%)	14 (82.4%)
No	78 (13.6%)	28 (9.7%)	45 (19.7%)	2 (4.9%)	3 (17.6%)

Among respondents, 356 (62%) were general pediatricians, and 218 (38%) were subspecialists, mainly in infectious diseases (N = 60, 27.5%), emergency medicine/general pediatrics (N = 31, 14.2%), and neonatology (N = 31, 14.2%) (S1 Table). Most worked in community settings (N = 297, 51.7%), while 103 (17.9%) and 175 (30.4%) were in primary/secondary and academic/tertiary hospitals, respectively. Urban/suburban practice was reported by 523 (91.3%). Over ten years of experience was noted in 422 (73.8%). Regarding bronchiolitis management, 277 (48.1%) “always” and 253 (43.9%) “very often” managed cases. Most (N = 496, 86.4%) worked in settings providing immunizations (Table 1).

Since 90% of respondents were from Spain and Italy, descriptive analyses and comparisons of RSV knowledge and prevention products are limited to these two countries. Data from Cyprus and Greece are reported as supplemental material. Hereafter, “overall” refers to the 518 participants from Spain and Italy. Among them, 212 fully completed the questionnaire. An internal comparison between respondents included in the main analysis and those with incomplete responses showed that the two groups were largely comparable across several key characteristics, although some differences in professional profile and practice setting were observed, particularly within individual countries (S2 Table).

### Knowledge of RSV and prevention products

Overall, most participants correctly identified prematurity (<32 gestational weeks: N = 427, 96.2%; 33–35 gestational weeks: N = 332, 74.8%), congenital heart disease (N = 387, 87.2%), chronic lung disease including bronchopulmonary dysplasia (N = 383, 86.3%), and Trisomy 21 (N = 243, 54.7%) as risk factors for severe RSV disease. Additionally, 253 (57.4%) participants identified healthy children under two years as being at the highest risk of severe RSV disease. Responses by country are shown in S1 and S2 Figs. Subspecialists were more likely to have medium-to-high RSV risk factors knowledge, while general pediatricians had lower scores (p = 0.054) (Table 2). In multivariable ordinal logistic regression analysis, some characteristics were associated with trends toward differences in the odds of being in a higher awareness category, with general pediatricians and those not providing immunizations showing lower odds, while pediatric subspecialists and those working in more specialized settings tended to have higher odds, although none of these associations reached statistical significance. Given the relatively small sample size and the distribution of responses across categories, the multivariable analyses were considered exploratory, and estimates should be interpreted with caution. In a stratified analysis stratified by pediatrician profile, some heterogeneity in awareness scores was observed across subgroups, with differences in score distribution by country and professional characteristics (S3 Table). These findings were exploratory and not formally tested for interaction.

**Table 2:** Level of knowledge of risk factors for severe RSV diseases among 212 respondents from Spain and Italy.

	Level (Score #1)				
	Low	Medium (N = 93)	High	P-value*	aOR [95% CI]
	(N = 34)		(N = 85)		
**Country**				0.292	
**Spain**	20 (58.8%)	50 (53.8%)	38 (44.7%)		–
**Italy**	14 (41.2%)	43 (46.2%)	47 (55.3%)		1.36 [0.79-2.32]
**Pediatric Subspecialty**				0.054	
**Pediatrician Subspecialist**	13 (38.2%)	39 (41.9%)	49 (57.7%)		–
**General Pediatrician**	21 (61.8%)	54 (58.1%)	36 (42.4%)		0.59 [0.21-1.62]
**Years in practice**				0.073	
**<=10**	9 (26.5%)	23 (24.7%)	34 (40.0%)		–
**> 10**	25 (73.5%)	70 (75.3%)	51 (60.0%)		0.69 [0.38-1.25]
**Description of primary practice**				0.063	
**Primary care&Primary outpatient center**	18 (52.9%)	53 (57.0%)	33 (38.8%)		–
**Primary/secondary care hospital&Private hospital**					
**Academic hospital&Tertiary care hospital**	2 (5.9%)	12 (12.9%)	18 (21.2%)		1.44 [0.43-4.84]
	14 (41.2%)	28 (30.1%)	34 (40.0%)		0.89 [0.31-2.55]
**Manage of RSV cases**				0.569	
**Always&Very often**	32 (94.1%)	85 (91.4%)	81 (95.3%)		–
**Sometimes&Rarely&Never**	2 (5.9%)	8 (8.6%)	4 (4.7%)		0.78 [0.27-2.22]
**Provision of immunizations to children**				0.120	
**Yes**					
**No**	26 (76.5%)	83 (89.3%)	76 (89.4%)		–
	8 (23.5%)	10 (10.8%)	9 (10.6%)		0.50 [0.23-1.11]

*Chi-square test was applied to assess the distribution of demographic and occupational characteristics among respondents with low, medium, or high levels of knowledge, treating these levels as independent variables.

Regarding knowledge of RSV prevention products, 178 (40.5%) respondents reported above average-to-very high knowledge of palivizumab, while 245 (55.9%) and 375 (86.0%) reported average-to-low knowledge of nirsevimab and the maternal RSV vaccine, respectively (S3 Fig). Perceived effectiveness of palivizumab was highest for reducing hospital admissions (N = 265, 72.0%), ICU admissions (N = 297, 81.3%), and mortality (N = 296, 83.3%) (S4 Fig).

Knowledge of prevention products was higher among Spanish pediatricians (p = 0.002), reflecting greater familiarity with nirsevimab in Spain, where it was already in use, and among subspecialists (p = 0.010) and those who work in academic/tertiary care hospitals (p = 0.023) (Table 3). In multivariable ordinal logistic regression analysis, pediatricians with more than 10 years of practice and those working in hospital or academic settings showed higher odds of being in a higher attitude category, while those practicing in Italy had lower odds compared with Spain. No meaningful differences were observed according to pediatric specialty, frequency of RSV case management, or provision of immunizations. These findings should be interpreted with caution given the study design and sample size.

**Table 3:** Level of knowledge of RSV prevention products (palivizumab, nirsevimab, and RSV maternal vaccine) among 212 respondents from Spain and Italy.

	Level (Score #2)				
	Low	Medium (N = 118)	High	P-value*	aOR [95% CI]
	(N = 18)		(N = 76)		
**Country**				**0.002**	
**Spain**	2 (11.1%)	62 (52.5%)	44 (57.9%)		–
**Italy**	16 (88.9%)	56 (47.5%)	32 (42.1%)		0.46 [0.26-0.83]
**Specialty**				**0.032**	
**Pediatrician Subspecialist**	6 (33.3%)	50 (42.4%)	45 (59.2%)		–
**General Pediatrician**	12 (66.7%)	68 (57.6%)	31 (40.8%)		0.95 [0.32-2.81]
**Years in practice**				0.085	
**<=10**	8 (44.4%)	41 (34.8%)	17 (22.4%)		–
**> 10**	10 (55.6%)	77 (65.3%)	59 (77.6%)		2.73 [1.43-5.20]
**Description of primary practice**				**0.023**	
**Primary care&Primary outpatient center**	12 (66.7%)	65 (55.1%)	27 (35.5%)		–
**Primary/secondary care hospital&Private hospital**					
**Academic hospital&Tertiary care hospital**	0 (0.0%)	17 (14.4%)	15 (19.7%)		4.54 [1.23-16.82]
	6 (33.3%)	36 (30.5%)	34 (44.7%)		2.65 [0.86-8.18]
**Manage of RSV cases**				0.797	
**Always&Very often**	17 (94.4%)	109 (92.4%)	72 (94.7%)		–
**Sometimes&Rarely&Never**	1 (5.6%)	9 (7.6%)	4 (5.3%)		0.83 [0.27-2.57]
**Provision of immunizations to children**				0.848	
**Yes**					
**No**	16 (89.9%)	104 (88.1%)	65 (85.5%)		–
	2 (11.1%)	14 (11.9%)	11 (14.5%)		1.17 [0.50-2.72]

*Chi-square test was applied to assess the distribution of demographic and occupational characteristics among respondents with low, medium, or high levels of knowledge, treating these levels as independent variables.

### Acceptance, attitude, practice, and perception of nirsevimab

Most respondents (N = 311, 85.9%) were likely to administer nirsevimab, with 319 (92.2%) indicating they would administer it to all patients, regardless of underlying risk factors (Tables 4 and S4). Notably, pediatricians with lower levels of knowledge were less likely to prescribe nirsevimab (S5 Table).

**Table 4:** Acceptance, attitude, and preference of respondents from Spain and Italy toward nirsevimab administration (N = 518).

Administration’s preference	N (%)
Likelihood of administering nirsevimab *(missing = 156)*	
Very likely	311 (85.9%)
Likely	39 (10.8%)
Neutral	9 (2.5%)
Unlikely	1 (0.3%)
Very unlikely	2 (0.6%)
Target population	
All infants (*missing = 172*)	319 (92.2%)
Only high-risk infants (*missing = 210)*	51 (16.6%)
*Only high-risk infants (n/51)*	
Preterm birth	51 (100.0%)
BDP/CLD	50 (98.0%)
CHD	49 (96.1%)
Trisomy 21	40 (78.4%)
Immunocompromised	46 (90.2%)
Infants lacking access to immediate medical care	26 (50.0%)
Other	2 (3.9%)
Preferred setting *(missing = 154)*	
During birth hospitalization	292 (80.2%)
During the first well-baby visit	33 (9.1%)
At a specific vaccine clinic	17 (4.7%)
At primary care centers	14 (3.8%)
Other	8 (2.2%)
Preferred time *(missing = 166)*	
Any time of the year	118 (33.5%)
Seasonal	234 (66.5%)

Regarding preferences, 175 (52.9%) respondents indicated a high likelihood of administering an RSV vaccine to infants over passive immunization, while 28.7% (N = 95) reported a moderate likelihood.

Birth hospitalization (N = 292, 80.2%) was the most preferred settings for nirsevimab administration. Notably, 118 (33.5%) participants favoured year-round over seasonal administration (Table 4).

**Fig 1:**
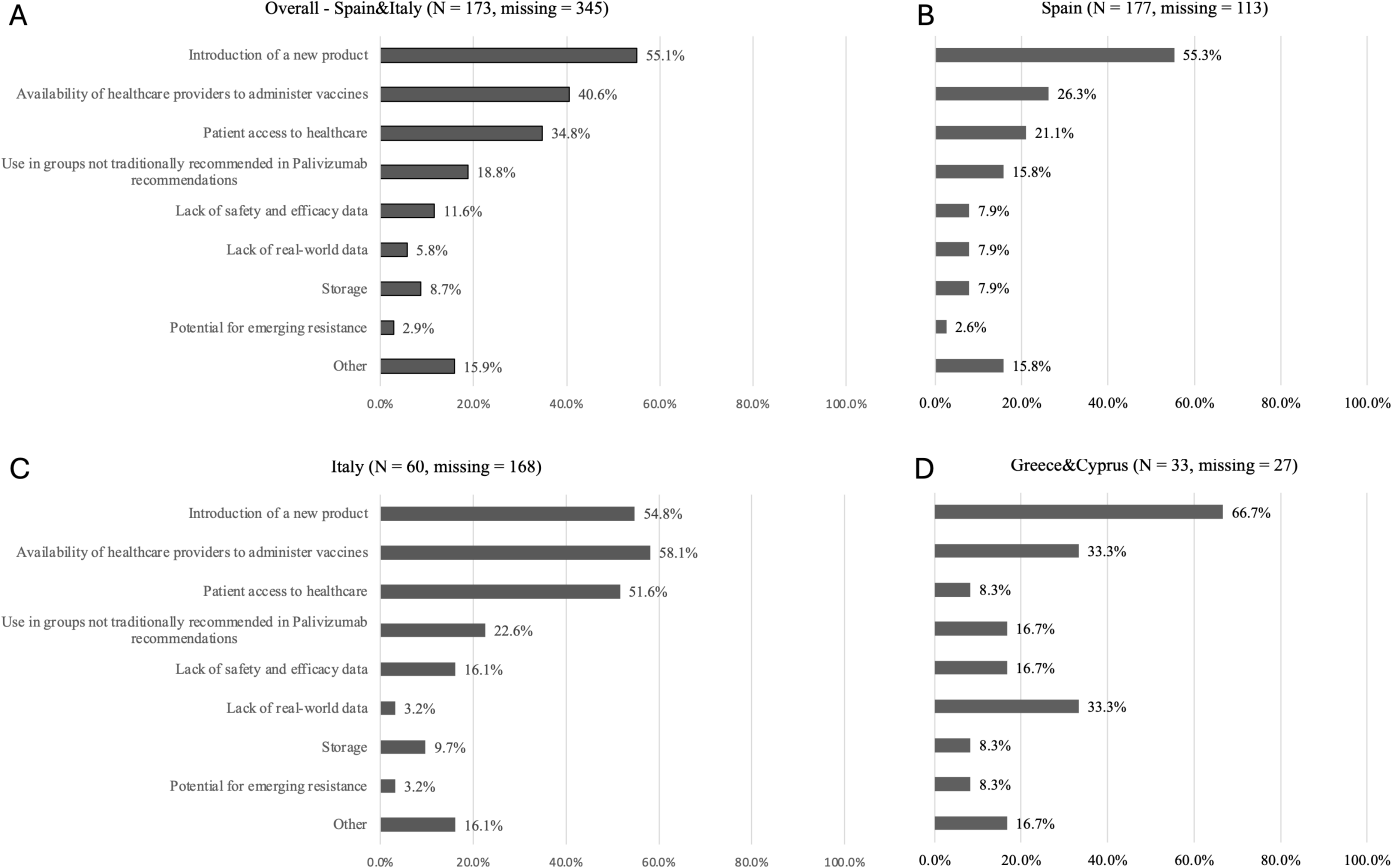
Specific barriers to nirsevimab administration identified among respondents by country.

Perceived barriers to nirsevimab administration were reported by 69 (20.0%) respondents, including concerns about introducing a new product (55.1%), healthcare provider availability (40.6%), and patient access (34.8%) (Fig 1). In terms of factors influencing pediatricians’ willingness to recommend nirsevimab, the most frequently cited were its demonstrated safety and real-world effectiveness (reported as “very important” by 87.7% and 89.7% of respondents, respectively), followed by trial efficacy (83.2%) and recommendations from agencies such as the ECDC (60.8%). Full details are provided in S6 Table.

Similarly, 118 (34.3%) respondents mentioned barriers to caregivers’ acceptance of nirsevimab, with the main concerns being caregivers’ perceptions about potential adverse effects (65.3%), lack of awareness about RSV (51.7%), and the administration of a monoclonal antibody to a healthy infant (44.1%) (S5 Fig).

### Acceptance, attitude, practices, and perception of RSV maternal vaccination

A total of 165 (47.4%) participants were more likely to recommend the RSV maternal vaccine over nirsevimab. However, 125 (38.5%) participants anticipated barriers to RSV maternal vaccine administration, with 60.8%, 58.4%, and 54.4% expressing concerns about introducing a vaccine during pregnancy, administering multiple vaccines during pregnancy, and potential adverse effects, respectively (S6 Fig). Pediatricians also cited key factors that would influence their recommendation of the maternal vaccine, including safety (89.4%), trial efficacy (84.4%), and real-world effectiveness (87.1%). National and international guidance, including from ECDC, was also considered “very important” by over 60% of respondents (S7 Table).

Additionally, 113 (32.6%) participants believed that acceptance of the RSV maternal vaccine would be lower or much lower than that of nirsevimab. A total of 145 (45.0%) anticipated barriers to caregivers’ acceptance of RSV maternal vaccine, with the most frequently mentioned concerns being caregivers’ perceptions of potential adverse effects on the infant (81.4%), potential adverse effects on the mother (75.9%), and lack of awareness about RSV (37.2%) (S7 Fig).

While 90 (27.4%) and 112 (34.0%) pediatricians routinely discussed influenza and Tdap vaccination with pregnant women, only 44 (13.4%) consistently addressed COVID-19 vaccination (S8 Fig). Nonetheless, 160 (47.8%) and 134 (40.0%) would “always” or “very often” recommend a multi-respiratory vaccine approach, including RSV, influenza, and COVID-19 vaccines.

## Discussion

RSV is a leading cause of acute respiratory infection and hospitalization in infants. Although two preventive strategies – maternal RSV vaccination and nirsevimab – are now recommended in many countries, real-world uptake depends on clinicians’ awareness, confidence, and implementation readiness. This multi-country survey assessed the attitudes, knowledge, and practices toward RSV immunization among 578 pediatricians from Spain, Italy, Cyprus, and Greece. Unlike previous single-country surveys, this study compares pediatricians in settings with active nirsevimab rollout versus those in a pre-implementation phase, offering a unique implementation-focused perspective.

Most participants expressed favorable attitudes toward nirsevimab, consistent with studies from Europe and the USA reporting high theoretical acceptance of RSV preventive products among pediatricians [29,30]. In our survey, 92.2% indicated they would administer nirsevimab to all infants, aligning with international recommendations [31]. The likelihood of prescribing nirsevimab increased with higher knowledge levels, reinforcing that education shapes clinical decision-making. Consistent with Riccò et al. [30], pediatricians with subspecialties demonstrated greater understanding of RSV risk factors, likely due to more frequent exposure to educational opportunities [29]. However, Riccò et al. assessed only knowledge in a single pre-implementation context [30]; our findings extend this by showing that knowledge translates into intended use only when supported by system readiness and product availability.

Our results expand and strengthen previous evidence. Papagiannis et al. found that Greek healthcare professionals recognized the importance of RSV immunization but lacked confidence in their knowledge and expressed a need for real-world data [32]. In Croatia, Mrcela et al. reported strong support for RSV prevention (71–76% agreed with monoclonal antibody use and 67–70% supported vaccination) [33], yet the study did not evaluate intention to use or implementation pathways, nor identify predictors of higher knowledge or likelihood of prescribing. Congedo et al. found limited awareness of nirsevimab and maternal vaccination among Italian pediatricians, with only 40% feeling confident in counseling caregivers [34]. A U.S. survey by Choi et al. similarly revealed a mismatch between awareness and willingness to recommend RSV immunization products [35]. Finally, the Turkish Pediatricians’ Atelier study found high awareness of nirsevimab (79.8%), but only 37.9% would administer it once available – compared with 92.2% in our study [36]. Taken together, these findings indicate that awareness and acceptance are necessary but not sufficient; clinical implementation requires confidence, training, and system support.

As expected, pediatricians in Italy – where nirsevimab was not yet introduced and national recommendation were not published – showed lower knowledge of nirsevimab than Spanish colleagues but were generally willing to prescribe it once available. This apparent willingness, despite limited knowledge, may reflect the hypothetical nature of questioning (“Once the product becomes available in your country, how likely are you to recommend it?”), but may also be informed by emerging trial results and early real-world data available at the time of the survey [8–19]. In addition, responses may be influenced by social desirability bias or implicit trust in future national recommendations. These findings highlight the importance of coupling policy implementation with targeted educational efforts to ensure informed clinical decision-making. Differences between Spain and Italy further support this interpretation, as pediatricians in Spain – where nirsevimab had already been implemented – showed higher familiarity and more consistent responses, suggesting that real-world experience may contribute to more grounded clinical decision-making compared with pre-implementation settings.

Unlike prior RSV surveys – which primarily focused on knowledge or theoretical acceptance – our study also evaluated implementation readiness, asking pediatricians to identify operational barriers (staffing, access, scheduling), immunization time preferences, and system-level needs. This provides a more pragmatic understanding of real-world adoption, highlighting that awareness and willingness to recommend nirsevimab are not sufficient unless supported by feasible implementation pathways and organizational capacity. Overall, 20.0% reported anticipated barriers to administration of nirsevimab, such as concerns about introducing a new product, limited healthcare provider availability, and restricted access. These barriers align with those identified by other authors, such as timing, familiarity, cost, reimbursement issues [37], and supply shortages [38]. These findings emphasize the need for strategies like targeted pediatrician training and enhanced logistical support to improve nirsevimab access. Pediatricians also anticipated caregivers’ barriers to acceptance of nirsevimab, with 65.3% mentioning concerns on caregivers’ perceptions about adverse effects, lack of RSV awareness, and hesitancy to administer monoclonal antibodies to healthy infants. These findings are consistent with previous studies showing that caregivers question nirsevimab’s safety and effectiveness [30], and low parental knowledge of RSV is a barrier to immunization acceptance at birth [39]. Since many caregivers plan vaccination schedules before pregnancy [40], providing early education on RSV during pregnancy could enhance nirsevimab acceptance and uptake. Moreover, a successful immunization program in Spain demonstrated that ensuring easy access to appointments – offered during non-working hours and at diverse locations – can significantly boost immunization rates [41].

Regarding immunization preferences, pediatricians favored administering nirsevimab during birth hospitalization or at the first well-baby visit. While most preferred seasonal administration, 33.5% expressed interest in year-round use. This preference may be particularly relevant for countries with limited primary care resources, as the long-acting nature of nirsevimab could facilitate its integration into routine visits or other immunization services. Such integration has previously been associated with higher vaccine uptake in resource-limited settings [42], as in low- and middle-income countries, common barriers to vaccination include distance to healthcare access points, lack of partner support, and inconvenient timing, which have been reported as key challenges for caregivers [43].

Despite 86.0% of respondents reporting an average-to-low level of knowledge of the RSV maternal vaccine, as well as evidence of its reduced efficacy [19], nearly half of participants would prefer administering the maternal vaccine over nirsevimab, potentially due to earlier introduction of national recommendations of maternal RSV vaccine in pregnancy in Italy. However, 32.6% believed that acceptance of the maternal vaccine would be lower than nirsevimab. About 60% of pediatricians reported regularly discussing maternal vaccinations against respiratory infections with pregnant women, despite evidence that recommending vaccines at each visit and emphasizing infant health is effective for maternal vaccination uptake [44]. This suggests a need for improved maternal vaccination education targeting healthcare providers who care for pregnant women and children.

This study has strengths, including geographic diversity and the inclusion of countries both with and without nirsevimab adoption. This design provides a broad evaluation of pediatricians’ attitudes and practices toward RSV prevention. The involvement of leading pediatric societies in each country further facilitated the inclusion of pediatricians who are actively engaged in RSV prevention efforts within their respective regions. However, the study has several limitations. The “self-administration” of participants may introduce bias, which could affect the generalizability of the results. The overall response rate of 19.3% is low. However, such rates are common in self-administered, non-incentivized surveys and are consistent with other online studies targeting healthcare professionals, including RSV-focused surveys in Greece (17.9%) and the United States (21%) [32,35]. Several factors may have contributed to the lower response, including the timing of distribution during July and August, and potential overlap with concurrent national surveys in Greece and Italy [32,34]. These competing demands may have limited participation, despite our efforts to engage members through different scientific societies than those involved in the other studies. This, in turn, resulted in a limited sample size, with only 10% of respondents from Cyprus and Greece. Consequently, the main analyses focused on data from Spain and Italy, while descriptive data from Cyprus and Greece are reported in the Online Supporting Resource. In addition, the substantial differences in response rates across countries may have influenced between-country comparisons, which should therefore be interpreted with caution. Furthermore, not all participants completed the full survey, potentially affecting the results. However, no meaningful differences were observed between overall respondents and those included in the main analysis, supporting the internal consistency of the findings. In addition, some heterogeneity in awareness levels was observed across pediatrician profiles, where country-level differences emerged. Although these findings are exploratory and were not formally tested for interaction, they may reflect underlying differences in training or clinical practice and should be interpreted with caution. This heterogeneity, together with the lack of information on the invited population, further limits the ability to assess the representativeness of the sample. The inclusion of uncompleted survey in the analysis is also a limitation. However, since the REDCap function that redirects users to their existing survey when they log in using the same email address was implemented, the risk of duplications and entry errors can be considered limited. Some biases may also arise due to the overrepresentation of specialists in infectious diseases, neonatology, and emergency medicine, who may be more likely to engage in topics related to RSV immunization. However, including neonatologists in the sample is crucial, as they are involved in administering monoclonal antibodies and play a key role in RSV prevention for infants. Moreover, the survey assessed pediatricians’ perceived barriers to the implementation of RSV immunization products, which may have led to a potentially inaccurate representation of caregiver-related barriers due to the lack of direct input from caregivers themselves. In addition, while the survey provides valuable insights, it is limited by its design compared to more robust epidemiological studies and ongoing research will be required as these new products are rolled out in different countries to understand the barriers and facilitators to real world implementation. Finally, the categorization of scores into low, medium, and high based on distribution percentiles is somewhat arbitrary and may influence the interpretation of results.

## Conclusions

Despite high acceptance of nirsevimab and the RSV maternal vaccine among European pediatricians, knowledge gaps and anticipated barriers to administration remain. Targeted educational initiatives to optimize pediatric practices regarding RSV infant immunization and maternal vaccination may be beneficial. Addressing these gaps could improve implementation and increase immunization rates, protecting vulnerable populations from RSV and other respiratory viruses.

## Supporting information

S1 File Survey instrument. (PDF)

S2 File Methods. (DOCX)

S1 Table Pediatric subspecialties of specialized participants (N = 218), overall and stratified by country. (DOCX)

S2 Table Baseline characteristics of respondents according to survey completion status in Spain and Italy. (DOCX)

S3 Table Baseline characteristics of respondents according to survey completion status in Spain and Italy. Distribution of awareness scores across pediatrician characteristics, stratified by professional training. Panel A, General Pediatrician; Panel B, Pediatrician subspecialist.
(DOCX)

S1 Fig Risk factors for severe RSV disease identified by 578 participants at the individual levels. Black bars represent correct answers, while gray bars indicate incorrect answers.
(DOCX)

S2 Fig Risk factors for severe RSV disease identified by 578 participants at the population levels. Black bars represent correct answers, while gray bars indicate incorrect answers.
(DOCX)

S3 Fig Perceived knowledge of previous (palivizumab) and new (nirsevimab and maternal vaccine) RSV prevention products. (DOCX)

S4 Fig Perceived effectiveness of palivizumab in preventing RSV infection outcomes. (DOCX)

S4 Table Acceptance, attitude, and preference of respondents toward nirsevimab administration (N = 578) by country. (DOCX)

S5 Table Knowledge of RSV risk factors (Score #1) and prevention products (Score #2: palivizumab, nirsevimab, maternal RSV vaccine) among Spanish and Italian pediatricians, by likelihood to prescribe nirsevimab and the maternal vaccine. (DOCX)

S6 Table Product characteristics and official recommendations influencing pediatricians’ willingness to recommend nirsevimab to parents. (DOCX)

S5 Fig Presence of perceived barriers to caregivers’ acceptance of nirsevimab and specific barriers identified among respondents. (DOCX)

S6 Fig Presence of perceived barriers to RSV maternal vaccine administration and specific barriers identified among respondents. (DOCX)

S7 Table Product characteristics and official recommendations influencing pediatricians’ willingness to recommend RSV maternal vaccine to pregnant women and families. (DOCX)

S7 Fig Presence of perceived barriers to RSV maternal vaccine acceptance and specific barriers identified among respondents. (DOCX)

S8 Fig Frequency with which respondents discuss influenza, COVID-19, and tetanus-diphtheria-pertussis vaccinations with pregnant women during their practice. (DOCX)
