# Supplementary material for: Pediatricians’ attitudes and knowledge of RSV immunization products: A multi-country cross-sectional survey
Source: PLoS One. 2026 Aug 17;21(8):e0356233. doi: 10.1371/journal.pone.0356233 (PMC13480629; doi:10.1371/journal.pone.0356233)
Supplement: Supplementary file 2 [file pone.0356233.s002.docx]

*Supplemental Materials. Methods*

Scores construction.

We developed three distinct scores to quantify (1) knowledge of RSV risk factors and (2) knowledge of RSV prevention products among participants. Higher scores indicate a greater level of knowledge.

Key questions were selected for each score to capture the most critical aspects of these areas.

Score #1: Knowledge of RSV risk factors

The questions *“At an individual level, which of the following underlying conditions increase the risk for severe RSV disease and are thus currently recommended to receive palivizumab/nirsevimab?”* (with *“palivizumab”* for Italy and *“nirsevimab”* for Spain) and *“At a population level, which group represents the largest proportion of severe RSV disease?”* were considered. The first question included nine evaluations, of which five were correct, and four were incorrect. The maximum possible score for this question was 9, with one point deduced for each incorrect classification. For the second question, one point was awarded if the response was correct. The first and second scores were summed, resulting in a total score #1 ranging from 5 to 10.

Score #2: Knowledge of RSV prevention products

This score was based on the question, *“On a scale from one (“very low”) to five (“very high”), how would you rate your knowledge of the following previous, current, and upcoming RSV prevention products?”*. The score was determined by asking participants to rate their knowledge of palivizumab, nirsevimab, and maternal vaccination on a scale of 1 (very low) to 5 (very high). Each product was assigned a score of 1-5. These three scores constituted the total score, ranging from a minimum of 3 to a maximum of 15.

The raw scores were standardized on a scale of 0 to 10. Subsequently, these normalized scores were categorized into three groups: 'low,' 'medium,' and 'high.' The cut-off points for these categories were determined by the 25th and 75th percentiles of the distribution of normalized scores. Given the study's primary objective of identifying pediatricians with lower levels of knowledge, the 'low' category was of particular.

SCORE2 min: 3 max: 15

normalization

SCORE2 min: 0 max: 10

categorization 25th pct: 3 75th pct: 6

low: 0-2 medium: 3-5 high: 6-10

SCORE1 min:5 max:10

normalization

SCORE1 min: 0 max: 10

categorization 25th pct: 4 75th pct: 8

low: 0-3 medium: 4-7 high: 8-10
