# Supplementary material for: Pediatricians’ attitudes and knowledge of RSV immunization products: A multi-country cross-sectional survey
Source: PLoS One. 2026 Aug 17;21(8):e0356233. doi: 10.1371/journal.pone.0356233 (PMC13480629; doi:10.1371/journal.pone.0356233)
Supplement: Supplementary file 3 [file pone.0356233.s003.docx]

*S1 Table.* Pediatric subspecialties of specialized participants (N=218), overall and stratified by country.

| Subspecialty N (%) | Overall (N=218) | Spain (N=108) | Italy  (N=97) | Cyprus (N=3) | Greece (N=10) |
| --- | --- | --- | --- | --- | --- |
| Allergy and immunology | 6 (2.8%) | 1 (0.9%) | 5 (5.2%) | 0 | 0 |
| Cardiology | 7 (3.2%) | 4 (3.7%) | 3 (3.1%) | 0 | 0 |
| Critical care | 16 (7.3%) | 10 (9.3%) | 5 (5.2%) | 0 | 1 (10%) |
| Dermatology | 1 (0.5%) | 0 | 1 (1%) | 0 | 0 |
| Emergency medicine and general pediatric ward | 31 (14.2%) | 19 (17.6%) | 12 (12.4%) | 0 | 0 |
| Endocrinology | 11 (5.1%) | 6 (5.6%) | 5 (5.2%) | 0 | 0 |
| Gastroenterology | 13 (6%) | 7 (6.5%) | 6 (6.2%) | 0 | 0 |
| Infectious diseases | 60 (27.5%) | 21 (19.4%) | 32 (33%) | 1 (33.4%) | 6 (60%) |
| Haematology/oncology | 14 (6.4%) | 7 (6.5%) | 6 (6.2%) | 0 | 1 (10%) |
| Nephrology | 12 (5.5%) | 7 (6.5%) | 5 (5.2%) | 0 | 0 |
| Neonatology | 31 (14.2%) | 18 (16.7%) | 10 (10.3%) | 0 | 3 (30%) |
| Neurology | 4 (1.8%) | 2 (1.9%) | 2 (2.1%) | 0 | 0 |
| Pulmonology | 9 (4.1%) | 5 (4.6%) | 3 (3.1%) | 1 (33.3%) | 0 |
| Rheumatology | 9 (4.1%) | 4 (3.7%) | 5 (5.2%) | 0 | 0 |
| Other | 13 (6%) | 12 (11.1%) | 1 (1%) | 0 | 0 |

Percentages do not sum to 100% as responses were not mutually exclusive.
