# Supplementary material for: Pediatricians’ attitudes and knowledge of RSV immunization products: A multi-country cross-sectional survey
Source: PLoS One. 2026 Aug 17;21(8):e0356233. doi: 10.1371/journal.pone.0356233 (PMC13480629; doi:10.1371/journal.pone.0356233)
Supplement: Supplementary file 4 [file pone.0356233.s004.docx]

*Table.S2.* Baseline characteristics of respondents according to survey completion status in Spain and Italy.

|  | Spain | | | Italy | | |
| --- | --- | --- | --- | --- | --- | --- |
| Characteristics, N (%) | **Complete respondents (N=108)** | **Incomplete respondents (N=182)** | **P-value*** | **Complete respondents (N=104)** | **Incomplete respondents (N=124)** | **P-value*** |
| Pediatric training |  | ***(missing=1)*** |  |  | ***(missing=3)*** |  |
| General pediatrics | 62 (57.4%) | 119 (65.8%) | 0.1563 | 49 (47.1%) | 79 (65.3%) | **0.0061** |
| Subspecialty pediatrics | 46 (42.6%) | 62 (34.3%) |  | 55 (52.9%) | 42 (34.7%) |  |
| Primary practice setting |  | ***(missing=1)*** |  |  | ***(missing=1)*** |  |
| Urban | 85 (78.7%) | 153 (84.5%) | 0.1307 | 80 (76.9%) | 92 (74.8%) | 0.2875 |
| Suburban | 9 (8.3%) | 17 (9.4%) |  | 18 (17.3%) | 17 (13.8%) |  |
| Rural/remote | 14 (13.0%) | 11 (6.1%) |  | 6 (5.8%) | 14 (11.4%) |  |
| Type of practice site |  | ***(missing=2)*** |  |  |  |  |
| Community-based pediatricians | 55 (50.9%) | 90 (50.0%) | 0.2737 | 49 (47.1%) | 81 (65.3%) | **0.0155** |
| Primary/secondary care hospital (public and private) | 11 (10.2%) | 30 (16.7%) |  | 21 (20.2%) | 20 (16.1%) |  |
| Academic/Tertiary care hospital | 42 (38.9%) | 60 (33.3%) |  | 34 (32.7%) | 23 (18.6%) |  |
| Years of practice |  | ***(missing=3)*** |  |  | ***(missing=3)*** |  |
| </= 5 years | 8 (7.4%) | 30 (16.8%) | **0.0415** | 27 (26.0%) | 19 (15.7%) | 0.1711 |
| 6-10 years | 18 (16.7%) | 19 (10.6%) |  | 13 (12.5%) | 11 (9.1%) |  |
| 11-25 years | 48 (44.4%) | 64 (35.8%) |  | 20 (19.2%) | 28 (23.1%) |  |
| >25 years | 34 (31.5%) | 66 (36.9) |  | 44 (42.3%) | 63 (52.1%) |  |
| Pediatricians’ practices in managing cases of bronchiolitis |  |  |  |  |  |  |
| Always | 60 (55.6%) | 91 (50.0%) | 0.7533 | 55 (52.9%) | 49 (39.5%) | 0.1469 |
| Very often | 41 (38.0%) | 77 (42.3%) |  | 42 (40.4%) | 65 (52.4%) |  |
| Sometimes | 6 (5.6%) | 13 (7.1%) |  | 5 (4.8%) | 9 (7.3%) |  |
| Rarely | 1 (0.9%) | 1 (0.6%) |  | 1 (1.0%) | 1 (0.8%) |  |
| Never | 0 (0.0%) | 0 (0.0%) |  | 1 (1.0%) | 0 (0.0%) |  |
| Working in a practice which provides immunizations |  | ***(missing=2)*** |  |  |  |  |
| Yes | 99 (91.7%) | 161 (89.4%) | 0.5377 | 86 (82.7%) | 97 (78.2%) | 0.3987 |
| No | 9 (8.3%) | 19 (10.6%) |  | 18 (17.3%) | 27 (21.8%) |  |

*Chi-square or Fisher’s exact test, as appropriate.
