# Supplementary material for: Pediatricians’ attitudes and knowledge of RSV immunization products: A multi-country cross-sectional survey
Source: PLoS One. 2026 Aug 17;21(8):e0356233. doi: 10.1371/journal.pone.0356233 (PMC13480629; doi:10.1371/journal.pone.0356233)
Supplement: Supplementary file 5 [file pone.0356233.s005.docx]

*Table.S3.* Baseline characteristics of respondents according to survey completion status in Spain and Italy. Distribution of awareness scores across pediatrician characteristics, stratified by professional training. Panel A, General Pediatrician; Panel B, Pediatrician subspecialist.

| Panel A | GENERAL PEDIATRICIAN  Level (Score #1) | | |  |
| --- | --- | --- | --- | --- |
|  | **Low**  **(N=21, 19%)** | **Medium**  **(N=54, 49%)** | **High**  **(N=36, 32%)** | **P-value*** |
| Country  Spain  Italy | 10 (47.6%)  11 (52.4%) | 30 (55.6%)  24 (44.4%) | 22 (61.1%)  14 (38.9%) | 0.6117 |
| Years in practice  <=10  >10 | 4 (19.1%)  17 (81.0%) | 9 (16.7%)  45 (83.3%) | 10 (27.8%)  26 (72.2%) | 0.4344 |
| Description of primary practice  Primary care&Primary outpatient center  Primary/secondary care hospital&Private hospital  Academic hospital&Tertiary care hospital | 18 (85.7%)  0 (0.0%)  3 (14.3%) | 49 (90.7%)  0 (0.0%)  5 (9.3%) | 33 (91.7%)  2 (5.6%)  1 (2.8%) | 0.1609 |
| Manage of RSV cases  Always&Very often  Sometimes&Rarely&Never | 20 (95.2%)  1 (4.8%) | 50 (92.6%)  4 (7.4%) | 35 (97.2%)  1 (2.8%) | 0.8551 |
| Provision of immunizations to children  Yes  No | 17 (81.0%)  4 (19.1%) | 50 (92.6%)  4 (7.4%) | 34 (94.4%)  2 (5.6%) | 0.2312 |

*Chi-square or Fisher’s exact test, as appropriate.

| Panel B | PEDIATRICIAN SUBSPECIALIST  Level (Score #1) | | |  |
| --- | --- | --- | --- | --- |
|  | **Low**  **(N=13, 13%)** | **Medium**  **(N=39, 39%)** | **High**  **(N=49, 48%)** | **P-value*** |
| Country  Spain  Italy | 10 (76.9%)  3 (23.1%) | 20 (51.3%)  19 (48.7%) | 16 (32.7%)  33 (67.4%) | **0.0113** |
| Years in practice  <=10  >10 | 5 (38.5%)  8 (61.5%) | 14 (35.9%)  25 (64.1%) | 24 (49.0%)  25 (51.0%) | 0.4441 |
| Description of primary practice  Primary care&Primary outpatient center  Primary/secondary care hospital&Private hospital  Academic hospital&Tertiary care hospital | 0 (0.00%)  2 (15.4%)  11 (84.6%) | 4 (10.3%)  12 (30.8%)  23 (59.0%) | 0 (0.0%)  16 (32.6%)  33 (67.4%) | 0.1119 |
| Manage of RSV cases  Always&Very often  Sometimes&Rarely&Never | 12 (92.3%)  1 (7.7%) | 35 (89.7%)  4 (10.3%) | 46 (93.9%)  3 (6.1%) | 0.8754 |
| Provision of immunizations to children  Yes  No | 9 (69.2%)  4 (30.8%) | 33 (84.6%)  6 (15.4%) | 42 (85.7%)  7 (14.3%) | 0.3704 |

*Chi-square or Fisher’s exact test, as appropriate.

| Panel C | GENERAL PEDIATRICIAN  Level (Score #2) | | |  |
| --- | --- | --- | --- | --- |
|  | **Low**  **(N=12)** | **Medium**  **(N=68)** | **High**  **(N=31)** | **P-value*** |
| Country  Spain  Italy | 1 (8.3%)  11 (91.7%) | 38 (55.9%)  30 (44.1%) | 23 (74.2%)  8 (25.8%) | **0.0005** |
| Years in practice  <=10  >10 | 3 (25.0%)  9 (75.0%) | 15 (22.1%)  53 (77.9%) | 5 (16.1%)  26 (83.9%) | 0.7387 |
| Description of primary practice  Primary care&Primary outpatient center  Primary/secondary care hospital&Private hospital  Academic hospital&Tertiary care hospital | 12 (100.0%)  0 (0.0%)  0 (0.0%) | 62 (91.2%)  0 (0.0%)  6 (8.8%) | 26 (83.9%)  2 (6.5%)  3 (9.7%) | 0.2619 |
| Manage of RSV cases  Always&Very often  Sometimes&Rarely&Never | 12 (100.0%)  0 (0.0%) | 62 (91.2%)  6 (8.8%) | 31 (100.0%)  0 (0.0%) | 0.2237 |
| Provision of immunizations to children  Yes  No | 11 (91.7%)  1 (8.3%) | 61 (89.7%)  7 (10.3%) | 29 (93.6%)  2 (6..5%) | 0.8855 |

*Chi-square or Fisher’s exact test, as appropriate.

| Panel D | PEDIATRICIAN SUBSPECIALIST  Level (Score #2) | | |  |
| --- | --- | --- | --- | --- |
|  | **Low**  **(N=6)** | **Medium**  **(N=50)** | **High**  **(N=45)** | **P-value*** |
| Country  Spain  Italy | 1 (16.7%)  5 (83.3%) | 24 (48.0%)  26 (52.0%) | 21 (46.7%)  24 (53.3%) | 0.4030 |
| Years in practice  <=10  >10 | 5 (83.3%)  1 (16.7%) | 26 (52.0%)  24 (48.0%) | 12 (26.7%)  33 (73.3%) | **0.0035** |
| Description of primary practice  Primary care&Primary outpatient center  Primary/secondary care hospital&Private hospital  Academic hospital&Tertiary care hospital | 0 (0.0%)  0 (0.0%)  6 (100.0%) | 3 (6.0%)  17 (34.0%)  30 (60.0%) | 1 (2.2%)  13 (28.9%)  31 (68.9%) | 0.3772 |
| Manage of RSV cases  Always&Very often  Sometimes&Rarely&Never | 5 (83.3%)  1 (16.7%) | 47 (94.0%)  3 (6.0%) | 41 (91.1%)  4 (8.9%) | 0.4659 |
| Provision of immunizations to children  Yes  No | 5 (83.3%)  1 (16.7%) | 43 (86.0%)  7 (14.0%) | 36 (80.0%)  9 (20.0%) | 0.7603 |

*Chi-square or Fisher’s exact test, as appropriate.
