# Supplementary material for: Pediatricians’ attitudes and knowledge of RSV immunization products: A multi-country cross-sectional survey
Source: PLoS One. 2026 Aug 17;21(8):e0356233. doi: 10.1371/journal.pone.0356233 (PMC13480629; doi:10.1371/journal.pone.0356233)
Supplement: Supplementary file 9 [file pone.0356233.s009.docx]

*S4 Fig.* Perceived effectiveness of palivizumab in preventing RSV infection outcomes.

ICU=intensive care unit.
