# Supplementary material for: Pediatricians’ attitudes and knowledge of RSV immunization products: A multi-country cross-sectional survey
Source: PLoS One. 2026 Aug 17;21(8):e0356233. doi: 10.1371/journal.pone.0356233 (PMC13480629; doi:10.1371/journal.pone.0356233)
Supplement: Supplementary file 10 [file pone.0356233.s010.docx]

*S4 Table. Table.S4.* Acceptance, attitude, and preference of respondents toward nirsevimab administration (N=578) by country.

| Administration’s preference | Spain  (N=290) | Italy  (N=228) | Greece&Cyprus  (N=60) |
| --- | --- | --- | --- |
| Likelihood of administering nirsevimab | *(missing=110)* | *(missing=46)* | *(missing=27)* |
| Very likely | 173 (96.1%) | 138 (75.8%) | 15 (45.5%) |
| Likely | 4 (2.2%) | 35 (19.2%) | 14 (42.4%) |
| Neutral | 0 (0.0%) | 9 (4.9%) | 2 (6.1%) |
| Unlikely | 1 (0.6%) | 0 (0.0%) | 1 (3%) |
| Very unlikely | 2 (1.1%) | 0 (0.0%) | 1 (3%) |
| Target population |  |  |  |
| All infants | 171 (96.1%)  *(missing=112)* | 148 (88.1%)  *(missing=60)* | 10 (33.3%)  *(missing=30)* |
| Only high-risk infants | 18 (11.8%)  *(missing=138)* | 33 (21.2%)  *(missing=72)* | 24 (72.7%)  *(missing=27)* |
| *Only high-risk infants* | *(n/18)* | *(n/33)* | *(n/24)* |
| Preterm birth | 18 (100.0%) | 33 (100.0%) | 23 (95.8%) |
| BDP/CLD | 17 (94.4%) | 33 (100.0%) | 22 (91.7%) |
| CHD | 18 (100.0%) | 31 (93.9%) | 20 (83.3%) |
| Trisomy 21 | 18 (100.0%) | 22 (66.7%) | 16 (66.7%) |
| Immunocompromised | 18 (100.0%) | 28 (84.9%) | 18 (75.0%) |
| Infants lacking access to immediate medical care | 8 (44.4%) | 18 (54.6%) | 8 (33.3%) |
| Other | 1 (5.6%) | 1 (3.0%) | 0 (0.0%) |
| Preferred setting | *(missing=108)* | *(missing=46)* | *(missing=27)* |
| During birth hospitalization | 164 (90.1%) | 128 (70.3%) | 14 (42.4%) |
| During the first well-baby visit | 4 (2.2%) | 29 (15.9%) | 12 (36.4%) |
| At a specific vaccine clinic | 0 (0.0%) | 17 (9.3%) | 6 (18.2%) |
| At primary care centers | 14 (7.7%) | 0 (0.0%) | 0 (0.0%) |
| Other | 0 (0.0%) | 8 (4.4%) | 1 (3%) |
| Preferred time | *(missing=112)* | *(missing=54)* | *(missing=27)* |
| Any time of the year | 47 (26.4%) | 71 (40.8%) | 8 (24.2%) |
| Seasonal | 131 (73.6%) | 103 (592%) | 25 (75.8%) |
