# Supplementary material for: Pediatricians’ attitudes and knowledge of RSV immunization products: A multi-country cross-sectional survey
Source: PLoS One. 2026 Aug 17;21(8):e0356233. doi: 10.1371/journal.pone.0356233 (PMC13480629; doi:10.1371/journal.pone.0356233)
Supplement: Supplementary file 11 [file pone.0356233.s011.docx]

*S5 Table*. Knowledge of RSV risk factors (Score #1) and prevention products (Score #2: palivizumab, nirsevimab, maternal RSV vaccine) among Spanish and Italian pediatricians, by likelihood to prescribe nirsevimab and the maternal vaccine.

|  | **Knowledge of RSV risk factors**  **(Score #1)** | | |  |
| --- | --- | --- | --- | --- |
|  | **Low**  **(N=34)** | **Medium**  **(N=93)** | **High**  **(N=85)** | |
| **Pediatricians’ likelihood of prescribing nirsevimab**  Likely  Neutral/unlikely | 33 (97.1%)  1 (2.9%) | 90 (96.8%)  3 (3.2%) | 82 (96.5%)  3 (3.5%) | |
| **Pediatricians’ likelihood of prescribing the RSV maternal vaccine**  Likely  Neutral/unlikely | 25 (73.5%)  9 (26.5%) | 69 (74.2%)  24 (25.8%) | 63 (74.1%)  22 (25.9%) | |
|  | **Knowledge of RSV prevention products**  **(Score #2)** | | |  |
|  | **Low**  **(N=18)** | **Medium**  **(N=118)** | **High**  **(N=76)** | |
| **Pediatricians’ likelihood of prescribing nirsevimab**  Likely  Neutral/unlikely | 17 (94.4%)  1 (5.6%) | 114 (96.6%) 4 (3.4%) | 74 (97.4%)  2 (2.6%) | |
| **Pediatricians’ likelihood of prescribing the RSV maternal vaccine**  Likely  Neutral/unlikely | 14 (77.8%)  4 (22.2%) | 90 (76.3%)  28 (23.7%) | 53 (69.7%)  23 (30.3%) | |
