# Supplementary material for: Pediatricians’ attitudes and knowledge of RSV immunization products: A multi-country cross-sectional survey
Source: PLoS One. 2026 Aug 17;21(8):e0356233. doi: 10.1371/journal.pone.0356233 (PMC13480629; doi:10.1371/journal.pone.0356233)
Supplement: Supplementary file 12 [file pone.0356233.s012.docx]

*S6 Table.* Product characteristics and official recommendations influencing pediatricians’ willingness to recommend nirsevimab to parents.

| Participants’ preferences | N (%) |
| --- | --- |
| Safety in clinical trial (missing=197, 34.08%)  Very important  Important  Neutral  Not very important  Not at all important  Efficacy in clinical trial (missing=198, 34.27%)  Very important  Important  Neutral  Not very important  Not at all important  Real-world effectiveness (missing=198, 34.27%)  Very important  Important  Neutral  Not very important  Not at all important  ECDC recommendation (missing=198, 34.27%)  Very important  Important  Neutral  Not very important  Not at all important  Italian pediatric society and Italian pediatric infectious diseases society (SITIP) (missing=403, 69.72%)^c^  Very important  Important  Neutral  Not very important  Not at all important  Ped Society position statement (missing=563, 97.40%)^d^  Very important  Important  Neutral  Not very important  Not at all important  Spanish Ministry of Health recommendations (missing= 401, 69.38%)^e^  Very important  Important  Neutral  Not very important  Not at all important  Asociación Española de Pediatría Sociedad Española de Infectología Pediátrica (AEP SEIP) position statement (missing=400, 69.20%)^e^  Very important  Important  Neutral  Not very important  Not at all important  Expert opinion (missing=202, 34.95%)  Very important  Important  Neutral  Not very important  Not at all important  Ease of administration and access (missing=197, 34.08%)  Very important  Important  Neutral  Not very important  Not at all important | 334 (87.7%)  40 (10.5%)  3 (0.8%)  3 (0.8%)  1 (0.3%)  316 (83.2%)  57 (15%)  3 (0.8%)  3 (0.8%)  1 (0.3%)  341 (89.7%)  33 (8.7%)  2 (0.5%)  3 (0.8%)  1 (0.3%)  231 (60.8%)  128 (33.7%)  17 (4.5%)  3 (0.8%)  1 (0.3%)  101 (57.7%)  65 (37.1%)  6 (3.4%)  3 (1.7%)  0  10 (66.7%)  4 (26.7%)  1 (6.7%)  0  0  97 (54.8%)  71 (40.1%)  8 (4.5%)  0  1 (0.6%)  129 (72.5%)  45 (25.3%)  3 (1.7%)  0  1 (0.6%)  144 (38.3%)  148 (39.4%)  69 (18.4%)  13 (3.5%)  2 (0.5%)  255 (66.9%)  111 (29.1%)  11 (2.9%)  3 (0.8%)  1 (0.3%) |
|  |  |
