# Supplementary material for: Pediatricians’ attitudes and knowledge of RSV immunization products: A multi-country cross-sectional survey
Source: PLoS One. 2026 Aug 17;21(8):e0356233. doi: 10.1371/journal.pone.0356233 (PMC13480629; doi:10.1371/journal.pone.0356233)
Supplement: Supplementary file 15 [file pone.0356233.s015.docx]

*Table.S7.* Product characteristics and official recommendations influencing pediatricians’ willingness to recommend RSV maternal vaccine to pregnant women and families.

| Participants’ preferences | N (%) |
| --- | --- |
| Safety in clinical trial (missing=209, 36.16%)  Very important  Important  Neutral  Not very important  Not at all important  Efficacy in clinical trial (missing=213, 36.85%)  Very important  Important  Neutral  Not very important  Not at all important  Real-world effectiveness (missing=214, 37.02%)  Very important  Important  Neutral  Not very important  Not at all important  ECDC recommendation (missing=212, 36.68%)  Very important  Important  Neutral  Not very important  Not at all important  SIP SITIP position statement (missing=414, 71.63%)^c^  Very important  Important  Neutral  Not very important  Not at all important  Ped Society position statement (missing=564, 97.58%)^d^  Very important  Important  Neutral  Not very important  Not at all important  Spanish Ministry of Health recommendations (missing=412, 71.28%)^e^  Very important  Important  Neutral  Not very important  Not at all important  AEP SEIP position statement (missing=410, 70.93%)^e^  Very important  Important  Neutral  Not very important  Not at all important  Expert opinion (missing=218, 37.72%)  Very important  Important  Neutral  Not very important  Not at all important  Ease of administration and access (missing=219, 37.89%)  Very important  Important  Neutral  Not very important  Not at all important | 330 (89.4%)  34 (9.2%)  3 (0.8%)  2 (0.5%)  0  308 (84.4%)  53 (14.5%)  2 (0.6%)  2 (0.6%)  0  317 (87.1%)  43 (11.8%)  2 (0.6%)  2 (0.6%)  0  237 (64.8%)  111 (30.3%)  16 (4.4%)  2 (0.6%)  0  90 (54.9%)  59 (36%)  10 (6.1%)  4 (2.4%)  1 (0.6%)  9 (64.3%)  3 (21.4%)  2 (14.3%)  0  0  99 (59.6%)  60 (36.1%)  6 (3.6%)  1 (0.6%)  0  121 (72%)  42 (25%)  4 (2.4%)  1 (0.6%)  0  138 (38.3%)  136 (37.8%)  68 (18.9%)  13 (3.6%)  5 (1.4%)  225 (62.7%)  115 (32%)  12 (3.3%)  5 (1.4%)  2 (0.6%) |
