# Supplementary material for: Pediatricians’ attitudes and knowledge of RSV immunization products: A multi-country cross-sectional survey
Source: PLoS One. 2026 Aug 17;21(8):e0356233. doi: 10.1371/journal.pone.0356233 (PMC13480629; doi:10.1371/journal.pone.0356233)
Supplement: Supplementary file 17 [file pone.0356233.s017.docx]

*S8 Fig*. Frequency with which respondents discuss influenza, COVID-19, and tetanus-diphtheria-pertussis vaccinations with pregnant women during their practice.
