# Supplementary material for: Pediatricians’ attitudes and knowledge of RSV immunization products: A multi-country cross-sectional survey
Source: PLoS One. 2026 Aug 17;21(8):e0356233. doi: 10.1371/journal.pone.0356233 (PMC13480629; doi:10.1371/journal.pone.0356233)
Supplement: Supplementary file 18 [file pone.0356233.s018.docx]

**Overall assessment and contribution**

This manuscript reports a cross sectional, self administered, multilingual survey of paediatricians in Spain, Italy, Cyprus, and Greece, conducted in July and August 2024 and distributed through scientific societies with weekly reminders. The topic is timely, given the rollout of nirsevimab and the introduction of maternal RSV vaccination. The manuscript adds an implementation perspective by exploring operational barriers and preferences regarding where and when these immunisation products should be administered.

**Major issues that should be addressed before the manuscript can be considered further**

**1) Representativeness and non response bias**

The overall response rate is low and highly heterogeneous across countries, with Spain much lower than Italy, plus limited participation from Cyprus and Greece, which led the authors to restrict the main analysis to Spain and Italy. This is a major limitation, since respondents may not reflect the paediatrician population in each country, or even the population of society members.

Actionable suggestions:
• Provide a more detailed comparison between respondents and the invited population, at least for any variables available through society rosters (for example, subspecialty, region, practice type), or explain why this was not feasible.
• Add a sensitivity analysis stratified by respondent profile, given potential over representation of subspecialists and hospital based clinicians.
• Discuss how the extreme between country differences in response rates may affect cross country comparisons.

**2) Analytical strategy relies mainly on univariable tests**

The manuscript is largely descriptive and uses simple association tests without multivariable adjustment. At the same time, parts of the text interpret relationships such as “higher knowledge increases intention”, which may be confounded by country, professional profile, and practice setting.

Actionable suggestions:
• Add multivariable models (for example, logistic regression) for key outcomes such as intention to administer nirsevimab, support for universal use, and preference for maternal vaccine versus nirsevimab. Candidate covariates already described include country, subspecialty, years in practice, practice setting, whether the service administers immunisations, and frequency of bronchiolitis management.
• Present effect estimates with confidence intervals (for example, odds ratios) rather than only p values.

**3) Handling of missingness and changing denominators**

The study retains incomplete questionnaires and computes proportions by question, excluding item level missing values. This approach can be acceptable, but it requires greater transparency regarding denominators, especially when missingness is high for central items.

Actionable suggestions:
• In all tables and figures, explicitly report the denominator analysed for each item, not only the total number per country.
• Add a parallel analysis restricted to fully completed questionnaires, at least for the main outcomes, since the knowledge scores were calculated only among those who answered all key questions.

**4) Construction and validation of knowledge scores**

The scores are described as aggregation of key variables, normalisation, and categorisation into low, medium, and high, with details in the supplement. This can be acceptable, but readers will need minimal information in the main text.

Actionable suggestions:
• In the main Methods, include a concise list of the variables included in each score and the rationale for any weighting (if applied) and for the cut points used to define low, medium, and high.
• Report internal consistency or at least a basic coherence check (for example, item to total correlation), even if exploratory.

**5) Interpretation given low familiarity with products**

An important finding is the high willingness to administer nirsevimab and support for universal use despite low self reported knowledge about nirsevimab and, especially, maternal vaccination. This is central to the conclusions, but the Discussion should explore alternative explanations more thoroughly, including social desirability bias and reliance on future recommendations.

Actionable suggestions:
• Make it explicit that this is stated intention in a hypothetical context, with a risk of overestimation.
• Where possible, contrast response patterns between Spain (where implementation occurred) and Italy (without implementation during the period) to show whether intention differs when practical experience exists.

**6) Data availability statement inconsistency with journal requirements**

There is a relevant inconsistency between the statement that “all relevant data are within the manuscript and supporting information” and another section stating that “data are available upon reasonable request.” For PLOS ONE, “available on request” is often insufficient unless strongly justified.

Actionable suggestions:
• Harmonise these statements and, ideally, deposit an anonymised dataset in a public repository, or clearly justify restrictions and specify an access pathway aligned with the journal’s policy.

**Minor but important points**

1. Timeline and national recommendation context
   The statement that only Spain had national recommendations during the study period is useful; consider highlighting this more prominently in the Introduction and at the start of the Results to guide the comparative reading.
2. Presentation of barriers
   Barriers to nirsevimab administration are presented as proportions among those who reported barriers and also as leading categories (new product introduction, staff availability, patient access). Please ensure the denominator is clearly stated in each figure and in the text.
3. Ethics and consent
   Ethics approval and the rationale for exemption in Spain and Italy due to anonymity are stated. Consider clarifying whether a formal waiver determination was obtained (even if not required) and how anonymity was ensured on the survey platform.

**Editorial recommendation**

Major revisions. The manuscript is relevant and contains interesting data, but it needs stronger adjusted analyses, clearer reporting of denominators and score construction, and resolution of the data availability issue to meet reproducibility expectations.
