# Supplementary material for: Pediatricians’ attitudes and knowledge of RSV immunization products: A multi-country cross-sectional survey
Source: PLoS One. 2026 Aug 17;21(8):e0356233. doi: 10.1371/journal.pone.0356233 (PMC13480629; doi:10.1371/journal.pone.0356233)
Supplement: Peer review history

# Peer review: Decision Letter 0

**DOI:** 10.1371/journal.pone.0356233.r001

**Authors:** Ricardo Gurgel

4 Apr 2026

Dear Dr. Di Chiara,

Please submit your revised manuscript by May 19 2026 11:59PM. If you will need more time than this to complete your revisions, please reply to this message or contact the journal office at plosone@plos.org. When you're ready to submit your revision, log on to https://www.editorialmanager.com/pone/ and select the 'Submissions Needing Revision' folder to locate your manuscript file.

- A letter that responds to each point raised by the academic editor and reviewer(s). You should upload this letter as a separate file labeled 'Response to Reviewers'.
- A marked-up copy of your manuscript that highlights changes made to the original version. You should upload this as a separate file labeled 'Revised Manuscript with Track Changes'.
- An unmarked version of your revised paper without tracked changes. You should upload this as a separate file labeled 'Manuscript'.

If you would like to make changes to your financial disclosure, please include your updated statement in your cover letter. Guidelines for resubmitting your figure files are available below the reviewer comments at the end of this letter.

If applicable, we recommend that you deposit your laboratory protocols in protocols.io to enhance the reproducibility of your results. Protocols.io assigns your protocol its own identifier (DOI) so that it can be cited independently in the future. For instructions see: https://journals.plos.org/plosone/s/submission-guidelines#loc-laboratory-protocols. Additionally, PLOS ONE offers an option for publishing peer-reviewed Lab Protocol articles, which describe protocols hosted on protocols.io. Read more information on sharing protocols at https://plos.org/protocols?utm_medium=editorial-email&utm_source=authorletters&utm_campaign=protocols.

As the corresponding author, your ORCID iD is verified in the submission system and will appear in the published article. PLOS supports the use of ORCID, and we encourage all coauthors to register for an ORCID iD and use it as well. Please encourage your coauthors to verify their ORCID iD within the submission system before final acceptance, as unverified ORCID iDs will not appear in the published article. *Only*  the individual author can complete the verification step; PLOS staff *cannot*  verify ORCID iDs on behalf of authors.

We look forward to receiving your revised manuscript.

Kind regards,

Ricardo Q. Gurgel, PhD

Academic Editor

PLOS One

Journal Requirements:

When submitting your revision, we need you to address these additional requirements.

1. Please ensure that your manuscript meets PLOS ONE's style requirements, including those for file naming. The PLOS ONE style templates can be found at

https://journals.plos.org/plosone/s/file?id=wjVg/PLOSOne_formatting_sample_main_body.pdf and https://journals.plos.org/plosone/s/file?id=ba62/PLOSOne_formatting_sample_title_authors_affiliations.pdf

2. Please include a complete copy of PLOS’ questionnaire on inclusivity in global research in your revised manuscript. Our policy for research in this area aims to improve transparency in the reporting of research performed outside of researchers’ own country or community. The policy applies to researchers who have travelled to a different country to conduct research, research with Indigenous populations or their lands, and research on cultural artefacts. The questionnaire can also be requested at the journal’s discretion for any other submissions, even if these conditions are not met. Please find more information on the policy and a link to download a blank copy of the questionnaire here: https://journals.plos.org/plosone/s/best-practices-in-research-reporting. Please upload a completed version of your questionnaire as Supporting Information when you resubmit your manuscript.

3. Your ethics statement should only appear in the Methods section of your manuscript. If your ethics statement is written in any section besides the Methods, please delete it from any other section.

4. We note that you have indicated that there are restrictions to data sharing for this study. For studies involving human research participant data or other sensitive data, we encourage authors to share de-identified or anonymized data. However, when data cannot be publicly shared for ethical reasons, we allow authors to make their data sets available upon request. For information on unacceptable data access restrictions, please see http://journals.plos.org/plosone/s/data-availability#loc-unacceptable-data-access-restrictions.

Before we proceed with your manuscript, please address the following prompts:

a) If there are ethical or legal restrictions on sharing a de-identified data set, please explain them in detail (e.g., data contain potentially identifying or sensitive patient information, data are owned by a third-party organization, etc.) and who has imposed them (e.g., a Research Ethics Committee or Institutional Review Board, etc.). Please also provide contact information for a data access committee, ethics committee, or other institutional body to which data requests may be sent.

b) If there are no restrictions, please upload the minimal anonymized data set necessary to replicate your study findings to a stable, public repository and provide us with the relevant URLs, DOIs, or accession numbers. Please see http://www.bmj.com/content/340/bmj.c181.long for guidelines on how to de-identify and prepare clinical data for publication. For a list of recommended repositories, please see https://journals.plos.org/plosone/s/recommended-repositories. You also have the option of uploading the data as Supporting Information files, but we would recommend depositing data directly to a data repository if possible.

Please update your Data Availability statement in the submission form accordingly.

5. Please amend your list of authors on the manuscript to ensure that each author is linked to an affiliation. Authors’ affiliations should reflect the institution where the work was done (if authors moved subsequently, you can also list the new affiliation stating “current affiliation:….” as necessary).

6. One of the noted authors is a group or consortium [Penta ID Respiratory Infection working group]. In addition to naming the author group, please list the individual authors and affiliations within this group in the acknowledgments section of your manuscript. Please also indicate clearly a lead author for this group along with a contact email address.

7. We note that Figure(s)  S1, in your submission contain [map/satellite] images which may be copyrighted. All PLOS content is published under the Creative Commons Attribution License (CC BY 4.0), which means that the manuscript, images, and Supporting Information files will be freely available online, and any third party is permitted to access, download, copy, distribute, and use these materials in any way, even commercially, with proper attribution. For these reasons, we cannot publish previously copyrighted maps or satellite images created using proprietary data, such as Google software (Google Maps, Street View, and Earth). For more information, see our copyright guidelines: http://journals.plos.org/plosone/s/licenses-and-copyright.

We require you to either (1) present written permission from the copyright holder to publish these figures specifically under the CC BY 4.0 license, or (2) remove the figures from your submission:

a. You may seek permission from the original copyright holder of Figure(s) S1 to publish the content specifically under the CC BY 4.0 license.

We recommend that you contact the original copyright holder with the Content Permission Form (http://journals.plos.org/plosone/s/file?id=7c09/content-permission-form.pdf) and the following text:

“I request permission for the open-access journal PLOS ONE to publish XXX under the Creative Commons Attribution License (CCAL) CC BY 4.0 (http://creativecommons.org/licenses/by/4.0/). Please be aware that this license allows unrestricted use and distribution, even commercially, by third parties. Please reply and provide explicit written permission to publish XXX under a CC BY license and complete the attached form.”

Please upload the completed Content Permission Form or other proof of granted permissions as an "Other" file with your submission.

In the figure caption of the copyrighted figure, please include the following text: “Reprinted from [ref] under a CC BY license, with permission from [name of publisher], original copyright [original copyright year].”

b. If you are unable to obtain permission from the original copyright holder to publish these figures under the CC BY 4.0 license or if the copyright holder’s requirements are incompatible with the CC BY 4.0 license, please either i) remove the figure or ii) supply a replacement figure that complies with the CC BY 4.0 license. Please check copyright information on all replacement figures and update the figure caption with source information. If applicable, please specify in the figure caption text when a figure is similar but not identical to the original image and is therefore for illustrative purposes only.

The following resources for replacing copyrighted map figures may be helpful:

USGS National Map Viewer (public domain): http://viewer.nationalmap.gov/viewer/

The Gateway to Astronaut Photography of Earth (public domain): http://eol.jsc.nasa.gov/sseop/clickmap/

Maps at the CIA (public domain): https://www.cia.gov/library/publications/the-world-factbook/index.html and https://www.cia.gov/library/publications/cia-maps-publications/index.html

NASA Earth Observatory (public domain): http://earthobservatory.nasa.gov/

Landsat: http://landsat.visibleearth.nasa.gov/

USGS EROS (Earth Resources Observatory and Science (EROS) Center) (public domain): http://eros.usgs.gov/#

Natural Earth (public domain): http://www.naturalearthdata.com/

8. If the reviewer comments include a recommendation to cite specific previously published works, please review and evaluate these publications to determine whether they are relevant and should be cited. There is no requirement to cite these works unless the editor has indicated otherwise.

[Note: HTML markup is below. Please do not edit.]

Reviewers' comments:

Reviewer's Responses to Questions

**Comments to the Author**

1. Is the manuscript technically sound, and do the data support the conclusions?

Reviewer #1: Yes

Reviewer #2: Yes

**********

2. Has the statistical analysis been performed appropriately and rigorously?

Reviewer #1: No

Reviewer #2: Yes

**********

3. Have the authors made all data underlying the findings in their manuscript fully available?

The PLOS Data policy

Reviewer #1: Yes

Reviewer #2: Yes

**********

4. Is the manuscript presented in an intelligible fashion and written in standard English?

Reviewer #1: Yes

Reviewer #2: Yes

**********

Reviewer #1: Major issues that should be addressed before the manuscript can be considered further

1) Representativeness and non response bias

The overall response rate is low and highly heterogeneous across countries, with Spain much lower than Italy, plus limited participation from Cyprus and Greece, which led the authors to restrict the main analysis to Spain and Italy. This is a major limitation, since respondents may not reflect the paediatrician population in each country, or even the population of society members.

Actionable suggestions:

• Provide a more detailed comparison between respondents and the invited population, at least for any variables available through society rosters (for example, subspecialty, region, practice type), or explain why this was not feasible.

• Add a sensitivity analysis stratified by respondent profile, given potential over representation of subspecialists and hospital based clinicians.

• Discuss how the extreme between country differences in response rates may affect cross country comparisons.

2) Analytical strategy relies mainly on univariable tests

The manuscript is largely descriptive and uses simple association tests without multivariable adjustment. At the same time, parts of the text interpret relationships such as “higher knowledge increases intention”, which may be confounded by country, professional profile, and practice setting.

Actionable suggestions:

• Add multivariable models (for example, logistic regression) for key outcomes such as intention to administer nirsevimab, support for universal use, and preference for maternal vaccine versus nirsevimab. Candidate covariates already described include country, subspecialty, years in practice, practice setting, whether the service administers immunisations, and frequency of bronchiolitis management.

• Present effect estimates with confidence intervals (for example, odds ratios) rather than only p values.

3) Handling of missingness and changing denominators

The study retains incomplete questionnaires and computes proportions by question, excluding item level missing values. This approach can be acceptable, but it requires greater transparency regarding denominators, especially when missingness is high for central items.

Actionable suggestions:

• In all tables and figures, explicitly report the denominator analysed for each item, not only the total number per country.

• Add a parallel analysis restricted to fully completed questionnaires, at least for the main outcomes, since the knowledge scores were calculated only among those who answered all key questions.

4) Construction and validation of knowledge scores

The scores are described as aggregation of key variables, normalisation, and categorisation into low, medium, and high, with details in the supplement. This can be acceptable, but readers will need minimal information in the main text.

Actionable suggestions:

• In the main Methods, include a concise list of the variables included in each score and the rationale for any weighting (if applied) and for the cut points used to define low, medium, and high.

• Report internal consistency or at least a basic coherence check (for example, item to total correlation), even if exploratory.

5) Interpretation given low familiarity with products

An important finding is the high willingness to administer nirsevimab and support for universal use despite low self reported knowledge about nirsevimab and, especially, maternal vaccination. This is central to the conclusions, but the Discussion should explore alternative explanations more thoroughly, including social desirability bias and reliance on future recommendations.

Actionable suggestions:

• Make it explicit that this is stated intention in a hypothetical context, with a risk of overestimation.

• Where possible, contrast response patterns between Spain (where implementation occurred) and Italy (without implementation during the period) to show whether intention differs when practical experience exists.

6) Data availability statement inconsistency with journal requirements

There is a relevant inconsistency between the statement that “all relevant data are within the manuscript and supporting information” and another section stating that “data are available upon reasonable request.” For PLOS ONE, “available on request” is often insufficient unless strongly justified.

Actionable suggestions:

• Harmonise these statements and, ideally, deposit an anonymised dataset in a public repository, or clearly justify restrictions and specify an access pathway aligned with the journal’s policy.

Reviewer #2: The research followed well-defined methodological criteria and achieved its objectives. It is important for the scientific community to note that there were conflicts of interest, which is pertinent and honest to acknowledge. Despite the industry's interest, the study is relevant and was approved by an ethics committee.

**********

what does this mean?). If published, this will include your full peer review and any attached files.

If you choose “no”, your identity will remain anonymous but your review may still be made public.

**Do you want your identity to be public for this peer review?**  For information about this choice, including consent withdrawal, please see our Privacy Policy

Reviewer #1: **Yes:**  OK

Reviewer #2: **Yes:**  Catia Maria Justo

**********

[NOTE: If reviewer comments were submitted as an attachment file, they will be attached to this email and accessible via the submission site. Please log into your account, locate the manuscript record, and check for the action link "View Attachments". If this link does not appear, there are no attachment files.]

To ensure your figures meet our technical requirements, please review our figure guidelines: https://journals.plos.org/plosone/s/figures

You may also use PLOS’s free figure tool, NAAS, to help you prepare publication quality figures: https://journals.plos.org/plosone/s/figures#loc-tools-for-figure-preparation.

NAAS will assess whether your figures meet our technical requirements by comparing each figure against our figure specifications.

Attachment Submitted filename: Overall assessment.docx

# Author comment: Author response to Decision Letter 1

**DOI:** 10.1371/journal.pone.0356233.r002

29 May 2026

Toronto, May 7, 2026

Dear Editor and Reviewers of the PLOS One journal,

We thank you for your interest and comments on our manuscript. We have addressed all comments in detail in this point-to-point letter.

We are pleased to resubmit our manuscript after revision, according to the Editor and Reviewers’ comments.

Costanza Di Chiara and Shaun K. Morris, on behalf of all authors

Reviewer #1:

Overall assessment and contribution

This manuscript reports a cross sectional, self administered, multilingual survey of paediatricians in Spain, Italy, Cyprus, and Greece, conducted in July and August 2024 and distributed through scientific societies with weekly reminders. The topic is timely, given the rollout of nirsevimab and the introduction of maternal RSV vaccination. The manuscript adds an implementation perspective by exploring operational barriers and preferences regarding where and when these immunisation products should be administered.

Major issues that should be addressed before the manuscript can be considered further

1-Representativeness and non response bias. The overall response rate is low and highly heterogeneous across countries, with Spain much lower than Italy, plus limited participation from Cyprus and Greece, which led the authors to restrict the main analysis to Spain and Italy. This is a major limitation, since respondents may not reflect the paediatrician population in each country, or even the population of society members.

Comment: Provide a more detailed comparison between respondents and the invited population, at least for any variables available through society rosters (for example, subspecialty, region, practice type), or explain why this was not feasible.

Reply: We thank the reviewer for this important comment. We acknowledge that the overall response rate was relatively low and heterogeneous across countries, and that this may limit the representativeness of the findings.

Unfortunately, we do not have access to detailed information on the full invited population (denominator), as the survey was disseminated through professional societies without individual-level data on all recipients. Therefore, a direct comparison between respondents and the invited population was not feasible.

To partially address this concern, we performed an internal comparison between respondents included in the main analysis (i.e., those with complete responses to the key survey panel) and those with incomplete responses, separately for Italy and Spain. This comparison is now reported in a new Supplemental Table.S2.

Overall, we observed that the two groups were largely comparable across several key characteristics, including practice setting, urban/rural distribution, and involvement in immunization activities. Some differences emerged, particularly in Italy (e.g., a higher proportion of subspecialists and hospital-based pediatricians among complete responders), suggesting a possible selection toward more specialized or academically oriented participants. In Spain, differences were less pronounced, although some variation in years of practice was observed.

These findings suggest that, while some degree of selection bias cannot be excluded, the population included in the main analysis does not appear to differ substantially from the broader group of respondents. Nevertheless, we have now explicitly acknowledged this limitation in the manuscript and clarified that the results may not be fully generalizable to the overall pediatrician population in each country.

Change: Supplemental materials, supplemental Table.S2

Comment: Add a sensitivity analysis stratified by respondent profile, given potential over representation of subspecialists and hospital based clinicians.

Reply: We explored whether pediatrician characteristics were associated with awareness levels. Overall, no consistent differences were observed among general pediatricians, who most frequently reported medium awareness scores. Among subspecialists, higher awareness levels were more commonly observed, with a predominance of high scores. However, country distribution differed significantly across score levels in this group, suggesting potential heterogeneity that warrants further investigation.

Change: Results, page 16: In a stratified analysis stratified by pediatrician profile, some heterogeneity in awareness scores was observed across subgroups, with differences in score distribution by country and professional characteristics (Supplementary Materials, Table.S3). These findings were exploratory and not formally tested for interaction.

Discussion, page 26: In addition, some heterogeneity in awareness levels was observed across pediatrician profiles, where country-level differences emerged. Although these findings are exploratory and were not formally tested for interaction, they may reflect underlying differences in training or clinical practice and should be interpreted with caution. This heterogeneity, together with the lack of information on the invited population, further limits the ability to assess the representativeness of the sample.

Supplemental materials, supplemental Table.S3

Comment: Discuss how the extreme between country differences in response rates may affect cross country comparisons.

Reply: We thank the reviewer for highlighting this important point. We agree that the marked heterogeneity in response rates across countries may limit the comparability of findings between settings. These differences likely reflect variations in survey dissemination, engagement, and participation within national societies, and may indicate that respondents differed across countries in their level of interest or involvement in this survey. Therefore, cross-country comparisons should be interpreted as exploratory rather than definitive.

Change: Discussion, page 26: In addition, the substantial differences in response rates across countries may have influenced between-country comparisons, which should therefore be interpreted with caution. Furthermore, not all participants completed the full survey, potentially affecting the results. However, no meaningful differences were observed between overall respondents and those included in the main analysis, supporting the internal consistency of the findings

2-Analytical strategy relies mainly on univariable tests

The manuscript is largely descriptive and uses simple association tests without multivariable adjustment. At the same time, parts of the text interpret relationships such as “higher knowledge increases intention”, which may be confounded by country, professional profile, and practice setting.

Comment: Add multivariable models (for example, logistic regression) for key outcomes such as intention to administer nirsevimab, support for universal use, and preference for maternal vaccine versus nirsevimab. Candidate covariates already described include country, subspecialty, years in practice, practice setting, whether the service administers immunisations, and frequency of bronchiolitis management.

Present effect estimates with confidence intervals (for example, odds ratios) rather than only p values.

Reply: We thank the reviewer for this valuable suggestion. In response, we have performed multivariable analyses to better account for potential confounding. Specifically, we fitted ordinal logistic regression models for the two main outcomes: Score 1 (awareness) and Score 2 (attitudes), including the covariates reported in Tables 2 and 3 (country, pediatrician profile, years in practice, practice setting, provision of immunizations, and frequency of bronchiolitis management). The corresponding adjusted odds ratios (ORs) with 95% confidence intervals have now been added directly to the tables.

We acknowledge that the confidence intervals are relatively wide, reflecting the limited sample size and the distribution of responses across categories. Therefore, these results should be interpreted with caution and considered exploratory.

Change: Methods, page 12: To further investigate associations between pediatrician characteristics and the main outcomes, multivariate analyses were conducted. Specifically, ordinal logistic regression models were fitted for awareness (Score 1) and attitudes (Score 2), both treated as ordered categorical variables (low, medium, high). Covariates included in the models were country, pediatrician profile, years in practice, primary practice setting, provision of immunizations, and frequency of bronchiolitis management. Covariates were selected a priori based on clinical relevance and prior literature on factors associated with knowledge and attitudes toward immunization. Results are reported as adjusted odds ratios (aORs) with 95% confidence intervals (CIs).

Results, pages 15–16: In multivariable ordinal logistic regression analysis, some characteristics were associated with trends toward differences in the odds of being in a higher awareness category, with general pediatricians and those not providing immunizations showing lower odds, while pediatric subspecialists and those working in more specialized settings tended to have higher odds, although none of these associations reached statistical significance. Given the relatively small sample size and the distribution of responses across categories, the multivariable analyses were considered exploratory, and estimates should be interpreted with caution.

Results, page 17: In multivariable ordinal logistic regression analysis, pediatricians with more than 10 years of practice and those working in hospital or academic settings showed higher odds of being in a higher attitude category, while those practicing in Italy had lower odds compared with Spain. No meaningful differences were observed according to pediatric specialty, frequency of RSV case management, or provision of immunizations. These findings should be interpreted with caution given the study design and sample size.

Table 2 and Table 3.

3-Handling of missingness and changing denominators

The study retains incomplete questionnaires and computes proportions by question, excluding item level missing values. This approach can be acceptable, but it requires greater transparency regarding denominators, especially when missingness is high for central items.

Comment: In all tables and figures, explicitly report the denominator analysed for each item, not only the total number per country.

Reply: We thank the reviewer for this comment. Table 1 has been revised to include the number of missing values for each specific variable evaluated. For Tables 2 and 3, by construction, no missing data are present, as these analyses were restricted to respondents with complete information for the variables included. In Table 4, variable-specific missing values had already been reported. Finally, in Figure 1, we have clarified the correct sample size, which serves as the denominator for each country-specific analysis.

Change: Table 1, and Figure 1.

Comment: Add a parallel analysis restricted to fully completed questionnaires, at least for the main outcomes, since the knowledge scores were calculated only among those who answered all key questions.

Reply: We thank the reviewer for this suggestion. The main analyses were already restricted to participants with complete responses for all items required to compute the knowledge and attitude scores. Among these 212 participants included in the analysis, the vast majority had near-complete questionnaires, with missing data limited to a small number of secondary or optional items (e.g., free-text “other” responses or less critical questions). We had initially chosen this approach to avoid excluding participants unnecessarily, particularly given the relatively small sample size. Restricting the analysis further to fully completed questionnaires would have led to the exclusion of only a very small number of additional participants (approximately 15), without materially affecting the results. For these reasons, we believe that an additional stratified analysis restricted to fully completed questionnaires would not provide meaningful additional insight. However, we have now clarified this point in the Methods section to improve transparency.

Change: Methods, page 12: Analyses were therefore restricted to this group, while minor missing data in secondary or optional variables were retained to avoid unnecessary reduction of the sample size.

4-Construction and validation of knowledge scores

The scores are described as aggregation of key variables, normalisation, and categorisation into low, medium, and high, with details in the supplement. This can be acceptable, but readers will need minimal information in the main text.

Comment: In the main Methods, include a concise list of the variables included in each score and the rationale for any weighting (if applied) and for the cut points used to define low, medium, and high.

Reply: We thank the reviewer for this suggestion, adding more details to the main text.

Change: Methods, page 11: Specific scores were developed to assess variations in RSV knowledge among pediatricians’ subgroups. These scores were constructed using a core set of key variables and designed to assess two dimensions: (1) knowledge of RSV risk factors and (2) knowledge of RSV prevention strategies. Higher scores indicated greater knowledge. The risk factor knowledge score (score #1) combined two components: identification of individual conditions associated with severe RSV disease (maximum 9 points, with penalties for incorrect responses) and identification of the population group accounting for the largest proportion of severe cases (1 point if correct), yielding a total score ranging from 5 to 10. The prevention knowledge score (score #2) was based on self-reported knowledge of three interventions (palivizumab, nirsevimab, and maternal vaccination), each rated on a 1–5 Likert scale, with a total score ranging from 3 to 15. Both scores were standardized to a 0–10 scale and categorized into three levels (low, medium, high) based on the 25th and 75th percentiles of their distribution to reflect relative differences within the study population. Additional details on score construction are provided in Supplementary Materials (Supplemental Methods).

Comment: Report internal consistency or at least a basic coherence check (for example, item to total correlation), even if exploratory.

Reply: We thank the reviewer for this suggestion. For the risk factor knowledge score (Score 1), formal internal consistency measures (e.g., Cronbach’s alpha) were not considered appropriate, as the score combines components with different structures and conceptual roles (a multi-item composite and a single binary item).

For the prevention knowledge score (Score 2), which includes multiple Likert-scale items, internal consistency could be assessed; however, given the exploratory nature of the study and the limited number of items, we prioritized a content-driven approach. Overall, score construction was guided by content validity and clinical relevance.

We have also acknowledged in the Discussion that the categorization of scores into low, medium, and high based on distribution percentiles is somewhat arbitrary and may influence the interpretation of the findings.

Change: Methods, page 11: The construction of the scores was guided by content validity and clinical relevance, and formal internal consistency measures were not considered appropriate given the structure and purpose of the scores.

Discussion, page 27: Finally, the categorization of scores into low, medium, and high based on distribution percentiles is somewhat arbitrary and may influence the interpretation of results.

5) Interpretation given low familiarity with products

An important finding is the high willingness to administer nirsevimab and support for universal use despite low self reported knowledge about nirsevimab and, especially, maternal vaccination. This is central to the conclusions, but the Discussion should explore alternative explanations more thoroughly, including social desirability bias and reliance on future recommendations.

Comment: Make it explicit that this is stated intention in a hypothetical context, with a risk of overestimation.

Reply: We thank the reviewer for

# Peer review: Decision Letter 1

**DOI:** 10.1371/journal.pone.0356233.r003

**Authors:** Ricardo Gurgel

3 Aug 2026

Pediatricians’ Attitudes and Knowledge of RSV Immunization Products: a multi-country cross-sectional survey

PONE-D-26-04721R1

Dear Dr. Di Chiara,

We’re pleased to inform you that your manuscript has been judged scientifically suitable for publication and will be formally accepted for publication once it meets all outstanding technical requirements.

Within one week, you’ll receive an e-mail detailing the required amendments. When these have been addressed, you’ll receive a formal acceptance letter and your manuscript will be scheduled for publication.

An invoice will be generated when your article is formally accepted. Please note, if your institution has a publishing partnership with PLOS and your article meets the relevant criteria, all or part of your publication costs will be covered. Please make sure your user information is up-to-date by logging into Editorial Manager at Editorial Manager® and clicking the ‘Update My Information' link at the top of the page. For questions related to billing, please contact billing support.

If your institution or institutions have a press office, please notify them about your upcoming paper to help maximize its impact. If they’ll be preparing press materials, please inform our press team as soon as possible -- no later than 48 hours after receiving the formal acceptance. Your manuscript will remain under strict press embargo until 2 pm Eastern Time on the date of publication. For more information, please contact onepress@plos.org.

Kind regards,

Ricardo Q. Gurgel, PhD

Academic Editor

PLOS One

Additional Editor Comments (optional):

Reviewers' comments:

Reviewer's Responses to Questions

**Comments to the Author**

Reviewer #1: All comments have been addressed

**********

2. Is the manuscript technically sound, and do the data support the conclusions?

Reviewer #1: Yes

**********

3. Has the statistical analysis been performed appropriately and rigorously?

Reviewer #1: Yes

**********

4. Have the authors made all data underlying the findings in their manuscript fully available?

The PLOS Data policy

Reviewer #1: Yes

**********

5. Is the manuscript presented in an intelligible fashion and written in standard English?

Reviewer #1: Yes

**********

Reviewer #1: (No Response)

**********

what does this mean?). If published, this will include your full peer review and any attached files.

If you choose “no”, your identity will remain anonymous but your review may still be made public.

**Do you want your identity to be public for this peer review?**  For information about this choice, including consent withdrawal, please see our Privacy Policy

Reviewer #1: **Yes:**  Eduardo Jorge da Fonseca Lima

**********

# Editor report: Acceptance letter

**DOI:** 10.1371/journal.pone.0356233.r004

**Authors:** Ricardo Gurgel

PONE-D-26-04721R1

PLOS One

Dear Dr. Morris,

I'm pleased to inform you that your manuscript has been deemed suitable for publication in PLOS One. Congratulations! Your manuscript is now being handed over to our production team.

At this stage, our production department will prepare your paper for publication. This includes ensuring the following:

* All references, tables, and figures are properly cited

* All relevant supporting information is included in the manuscript submission,

* There are no issues that prevent the paper from being properly typeset

You will receive further instructions from the production team, including instructions on how to review your proof when it is ready. Please keep in mind that we are working through a large volume of accepted articles, so please give us a few days to review your paper and let you know the next and final steps.

Lastly, if your institution or institutions have a press office, please let them know about your upcoming paper now to help maximize its impact. If they'll be preparing press materials, please inform our press team within the next 48 hours. Your manuscript will remain under strict press embargo until 2 pm Eastern Time on the date of publication. For more information, please contact onepress@plos.org.

You will receive an invoice from PLOS for your publication fee after your manuscript has reached the completed accept phase. If you receive an email requesting payment before acceptance or for any other service, this may be a phishing scheme. Learn how to identify phishing emails and protect your accounts at https://explore.plos.org/phishing.

If we can help with anything else, please email us at customercare@plos.org.

Thank you for submitting your work to PLOS ONE and supporting open access.

Kind regards,

PLOS ONE Editorial Office Staff

on behalf of

Professor Ricardo Q. Gurgel

Academic Editor

PLOS One
